# The Role of Microenvironmental Cues and Mechanical Loading Milieus in Breast Cancer Cell Progression and Metastasis

**DOI:** 10.3389/fbioe.2020.608526

**Published:** 2021-01-18

**Authors:** Brandon D. Riehl, Eunju Kim, Tasneem Bouzid, Jung Yul Lim

**Affiliations:** ^1^Department of Mechanical and Materials Engineering, University of Nebraska-Lincoln, Lincoln, NE, United States; ^2^Mary and Dick Holland Regenerative Medicine Program, University of Nebraska Medical Center, Omaha, NE, United States

**Keywords:** breast cancer, metastasis, substrate microenvironments, mechanical loading, mechanotransduction, cell progression

## Abstract

Cancer can disrupt the microenvironments and mechanical homeostatic actions in multiple scales from large tissue modification to altered cellular signaling pathway in mechanotransduction. In this review, we highlight recent progresses in breast cancer cell mechanobiology focusing on cell-microenvironment interaction and mechanical loading regulation of cells. First, the effects of microenvironmental cues on breast cancer cell progression and metastasis will be reviewed with respect to substrate stiffness, chemical/topographic substrate patterning, and 2D vs. 3D cultures. Then, the role of mechanical loading situations such as tensile stretch, compression, and flow-induced shear will be discussed in relation to breast cancer cell mechanobiology and metastasis prevention. Ultimately, the substrate microenvironment and mechanical signal will work together to control cancer cell progression and metastasis. The discussions on breast cancer cell responsiveness to mechanical signals, from static substrate and dynamic loading, and the mechanotransduction pathways involved will facilitate interdisciplinary knowledge transfer, enabling further insights into prognostic markers, mechanically mediated metastasis pathways for therapeutic targets, and model systems required to advance cancer mechanobiology.

## Introduction

Breast cancer is the most diagnosed cancer among women in the US with significant mortality resulting from metastasis (DeSantis et al., 2019). Early detection and prevention of breast cancer metastasis could greatly improve patient outcomes especially for cases when breast cancer migrates to crucial organs including brain, liver, lung, and bone. Recently, mechanical factors are becoming more and more recognized as important regulators of oncogenesis, tumor progression, and metastasis. The mechanical factors may originate from microenvironments, e.g., extracellular matrix (ECM) stiffness, topography, composition, etc., as well as from dynamic mechanical loading situations, e.g., tensile strain, compression, flow-induced shear, etc. Microenvironmental factors at the premetastatic niche may prime tumor cells toward invasive phenotypes causing a host of genetic and mechanical adaptations, which then can feedback to reinforce the aberrant ECM environment. Further adaptations accrue when tumor cells proliferate in a confined and stiffened ECM milieu causing a rise in solid stress within the tumor. As the vasculature in the tumor is remodeled, cells also experience elevated interstitial pressure and thus compressive effect. Moreover, tensile stress may be experienced at the tumor periphery, and interstitial outflow is becoming developed. In the next step, metastasizing cells are exposed to fluid flow-induced shear stress during distal migration through the lymph and vascular systems. Tumor cell migration through capillaries and intravasation and extravasation induce compression and large cellular deformations in the form of constrictions. Finally, each metastasis target site has unique microenvironmental and dynamic mechanical loading signals which may contribute to disease progression or induce dormancy. Here, we will first review the updated understanding on the effects of key microenvironmental cues on breast cancer cell behavior (the representative findings on microenvironments are highlighted in Table 1). The effects of dynamic mechanical loading environments on breast cancer cells will then be described including those at the distal metastasis site (representative findings on mechanical loading cues are highlighted in Table 2). Then, the discussion of potential regulatory cellular mechanotransduction mechanisms involved in the mechanical factor regulation of breast cancer cells is provided before ending with a perspective. Note, for detailed classification of breast cancer cell lines described in this review, the works by Neve et al. (2006) and Holliday and Speirs (2011) are referred.

**Table 1:** Some representative concepts/findings in microenvironmental cues.

**Section**	**References**	**Key concepts**
Stiffness	Plodinec et al., 2012	AFM performed on biopsies of benign and cancerous breast tissue reveals molecular and structural changes in both ECM and cells.
Stiffness	Omidvar et al., 2014	Cell stiffness is correlated with invasive properties with a converse correlation between the adhesion force between breast cancer cells and their invasive potential.
Stiffness	Yang et al., 2020	Rigidity sensing is impaired in cancer cells but remains active in normal mammary cells. Restoring cytoskeletal proteins rescued cells from cancerous responses with changes to local membrane contractions, adhesion, cytoskeletal organization, and inhibition of tumor formation.
Stiffness	Nasrollahi et al., 2017	Benign MCF-10A and cancerous MCF-7 cells have mechanical memory of substrate stiffness influencing later migration. Cells primed on a stiff substrate and later moved to a soft gel had faster migration, larger focal adhesions, and higher actomyosin expression compared to cells primed on a soft matrix. The memory is dependent on YAP nuclear transfer on stiff substrates.
Stiffness	Stowers et al., 2016	Benign epithelial cells grown in soft hydrogel formed healthy acini and exhibited quiescence. When the gels were stiffened to mimic tumor stiffness, this caused cell proliferation, Ki-67 expression, and an invasion to the surrounding gels away from the acini.
Surface patterns	Zhang and Webster, 2012	Malignant MCF-7 cells had significantly decreased VEGF synthesis with lower proliferation and increased apoptosis on 23 nm topographic features, whereas benign epithelial cells had increased proliferation on the topography.
Surface patterns	Chen et al., 2019	Cell migration direction is biased with sawtooth nanoscale ridges. MDA-MB-231 preferentially moved up the sawtooth formations, while M4 metastatic variant of MCF-10A moved in the opposite direction.
Surface patterns	Tseng et al., 2011	Micropatterning is used to assess cytoskeletal stress and arrangement and infer cell state and malignant status. For benign epithelial MCF-10A cells patterned on fibronectin crossbow, disc, and pacman shapes, all transformative tumor characteristics are not entirely involved with increased cell contractility.
Surface patterns	Tse et al., 2012	Migration leader cells were produced at sharp corners of micropatterns. Compressing the micropatterns created leader cells at the pattern boundary. Inhibition of ROCK or MLCK cytoskeletal regulator diminished the overall migration but did not prevent the formation of leader cells.
2D vs. 3D	Balachander et al., 2015	MDA-MB-231 cells grown in 3D scaffolds induced changes in invasive membrane structures and genes related to cancer stemness, inflammation, and cell-cell and cell-matrix adhesion compared to those on 2D surfaces. Cells from 3D culture developed larger tumors when implanted in the mammary fat pad.
2D vs. 3D	Matthews et al., 2020	Ras oncogene promotes cell rounding in confined environments which may promote cancer cell survival in compressive tumors due to less chromosome segregation errors.
2D vs. 3D	Stowers et al., 2019	Stiff 2D culture environments may cause loss of tissue function due to changes in chromatin. The epigenetic profile is sensitive to both dimensional and stiffness effects.

**Table 2:** Some representative concepts/findings in mechanical loading milieus.

**Section**	**References**	**Key concepts**
Stretch	Ansaryan et al., 2019	Stretching of cancerous cells causes increase in invadopodia, activation of aerobic glycolysis, and altered membrane voltage, all indicative of cancer progression. Benign MCF-10A, which underwent apoptosis in the static condition, could diffuse through the endothelial layer after stretch.
Stretch	Wang et al., 2020	Tensile stretching may activate motility pathways to increase transmigration of MCF-7, MDA-MB-231, and 4T1.2 cells. Stretch conditioned cells also modify local immune environment *via* exosome signaling which suppressed anti-tumorigenic immune response.
Stretch	Berrueta et al., 2016	Local stretching of tissue *in vivo* can reduce inflammation, fibrosis, and tumor volume. Mice implanted with breast cancer subjected to 10 min of forelimb to tail stretching had 52% smaller tumor compared to unstretched control mice.
Compression	Tien et al., 2012	Mechanical stress signals, the local ECM environment, and chemical factors are interconnected in tumor outgrowth. Pressure on one side of a 3D MDA-MB-231 aggregate inhibits tumor outgrowth from the opposite side potentially due to altered chemical microenvironment.
Compression	Ficorella et al., 2019	Invasion through ECM, intravasation, and extravasation induce compressive strains *via* constrictions on migrating cells. Mesenchymal-like MDA-MB-231 uses blebs to pass through the constriction, whereas the MCF-10A primarily uses lamellipodia with some blebbing.
Compression	Kim et al., 2019	Compression may contribute to tumor cell survival in hypoxic conditions by activating glycolysis genes and adapting cell metabolism and miRNA. Metabolic, EMT-related, and angiogenesis genes are upregulated in compressed cancer-associated fibroblasts compared to static control.
Compression	Fan et al., 2020	Regulation of tumor development by compression *in vivo* may depend on the loading magnitude. Loading mouse tibia with 1 N reduces bone destruction by tumor activity, while 5 N induces osteolysis with significant bone loss.
Fluid shear	Polacheck et al., 2011, 2014	MDA-MB-231 cells have heterogeneous migration responses to microfluidic interstitial flows depending on dimensionality, matrix material, cell density, flow velocity, and cell receptor activity. Cell adaptation *via* β1 integrin and paxillin focal adhesion has a key role, e.g., when paxillin was inhibited, MDA-MB-231 cells no longer migrate against the flow.
Fluid shear	Haessler et al., 2012	Fluid shear increases breast cancer cell motility in a 3D environment in a heterogeneous manner, implying simple averages of cell behavior might not reveal an accurate picture of migration.
Fluid shear	Riehl et al., 2020	Cells with higher metastatic potential (MDA-MB-231) display greater sensitivity in migration to fluid shear. Less metastatic MDA-MB-468 is less responsive to flow shear, and benign MCF-10A has the lowest migration potential under shear.
Fluid shear	Choi et al., 2019	Flow shear may promote the EMT process and render cancer cells to be more aggressive by activating embryonic-like stem properties through the deactivation of ERK and GSK3β.
Fluid shear	Zhang et al., 2018	MDA-MB-231cells injected to a mouse model from a suspension condition have significant increase in metastasis to the lungs compared with cells that are grown in an adhesive environment.
Fluid shear	Novak et al., 2019; Triantafillu et al., 2019	Fluid flow induced shear stress conditions may result in chemoresistance, e.g., to the drug paclitaxel and doxorubicin.

## Effects of Microenvironmental Cues

### Substrate Stiffness

It has been recognized that the composition, mechanical stiffness, and dimensionality of cancerous extracellular environments greatly influence cancer cell physiology and progression. Specifically, the alteration in tissue stiffness indicates one of the earliest indicators of cancer presence. Forces between cells and cell and ECM are dependent on the increase in breast tissue stiffness which is associated with the risk of breast cancer. This may be assessed radiologically with dense breast tissue having an increase in cell number, collagen fibers, and proteoglycans compared to breast tissue with low mammographic density (Boyd et al., 2014). Cancers have molecular and structural changes in both the ECM and cells. Such alteration is evident in atomic force microscopy (AFM) performed on biopsies of benign and cancerous breast tissues (Plodinec et al., 2012). Tissues from benign biopsies had uniform stiffness with a single peak in the correlative stiffness map, while malignant tissues showed heterogeneous stiffness with a broad distribution in correlative stiffness maps and a low-stiffness peak due to soft cancer cells. The differences in normal, tumorigenic, and metastatic cells have been demonstrated in another study by microrheology confirming cancerous cells are significantly softer than benign counterparts (Smelser et al., 2015). The surrounding tissues remodel and adapt in response to cancer growth resulting in aberrant ECM moduli. This can then work as a feedback loop since cancerous cells on abnormal stiffness have responses that may further cancer progression.

The changes in tissue stiffness have been correlated with breast cancer invasion and aggression. When cancer transforms the ECM, additional collagen is deposited and the local ECM at the tumor invasive front is linearized. Correlating this to invasive cell behavior, the ECM was stiffest and most heterogeneous around the aggressive basal-like, HER2+ tumors compared with less aggressive luminal tumors (Acerbi et al., 2015). The stiff matrix can cause alterations in cell adhesion structures and dynamics. For instance, the stiff matrix may induce a mesenchymal-like cell phenotype which is evident in the focal adhesion assemblies and forces produced, and such changes may be key to cancer cell invasion and migration in confined spaces (Mekhdjian et al., 2017). Interfaces to the cells are also altered in cancer with significant changes to glycocalyx cell coating in tumor cells contributing to malignancy. The glycocalyx coating can amplify mechanical signals on the cell surface and modify transmembrane protein expressions. For example, the expression of transmembrane mechanical linkage protein, integrin, is altered in cancer cells when large glycoproteins are present in the glycocalyx (Paszek et al., 2014). These stiffness adaptations can have, eventually, implications for the efficacy of cancer treatment. The efficiency of the therapeutic lapatinib which acts on the HER2 pathway in cancer cells was found to be modulated by microenvironment mechanical properties: HCC1569 breast cancer cells were responsive to lapatinib on a collagen gel with 400 Pa stiffness but this response was absent on the tissue culture plastic with >2 GPa stiffness (Lin et al., 2015).

In addition to altered ECM stiffness, as mentioned above, cancer cells themselves can also have altered stiffness. A converse correlation was found between the stiffness of the cancer cells and their invasive properties: in the order of invasiveness from less to more, the average stiffness as measured by AFM was 1.05, 0.94, and 0.62 kPa for MCF-7, T47D, and MDA-MB-231 cells, respectively (Omidvar et al., 2014). It has been established that breast cancer cells cultured on stiff surfaces have in general higher proliferation, higher migration velocity, and increased chemoresistance compared with those grown on soft substrates (McGrail et al., 2015). Furthermore, breast cancer cells showed a preference for stiffer surfaces by exhibiting durotaxis (i.e., migration from soft to stiff region) on polyacrylamide stiffness gradients (DuChez et al., 2019). Cells showed durotaxis with migration distance dependent on wherein the cells were seeded: cells starting in softer regions migrated further than cells seeded on medium or stiff regions of the gradient. This provides evidence that cancer cells can sense variations in the ECM stiffness as may be found in heterogeneous tumors or at tumor boundaries.

Via testing the deformation of sub-μm scale polydimethylsiloxane (PDMS) micropillars by the cells, it was found that substrate rigidity sensing was impaired in metastatic MDA-MB-231 cells but remained active in normal MCF-10A mammary cells (Wolfenson et al., 2016). As a mechanism, the rigidity sensing could be restored *via* tropomyosin 2.1 expression, or the normal cells could transform to a cancerous phenotype with tropomyosin 2.1 inhibition. In a follow up study (Yang et al., 2020), restoring cytoskeletal proteins rescued cells from cancerous responses with changes to local membrane contraction, adhesion, cytoskeletal organization, and as a result inhibited tumor formation. Moreover, inhibiting cytoskeletal elements including myosin IIA reinforced cancer phenotypes. Translating these results into an *in vivo* model, MDA-MB-231 cells with rigidity sensing restored by YFP-tropomyosin 2.1 transfection had less tumorigenic responses in a chick chorioallantoic model compared to green fluorescent protein (GFP) transfected control. The MDA-MB-231 cells with tropomyosin expression formed tumors with 36.5% lower tumor weight and the tumor volume decreased over time, whereas the control MDA-MB-231 cells had increased tumor volume with time. Taken together, these results demonstrate that cancerous cells have lower stiffness and fewer rigidity sensing mechanisms compared to normal cells.

**Figure 1:**
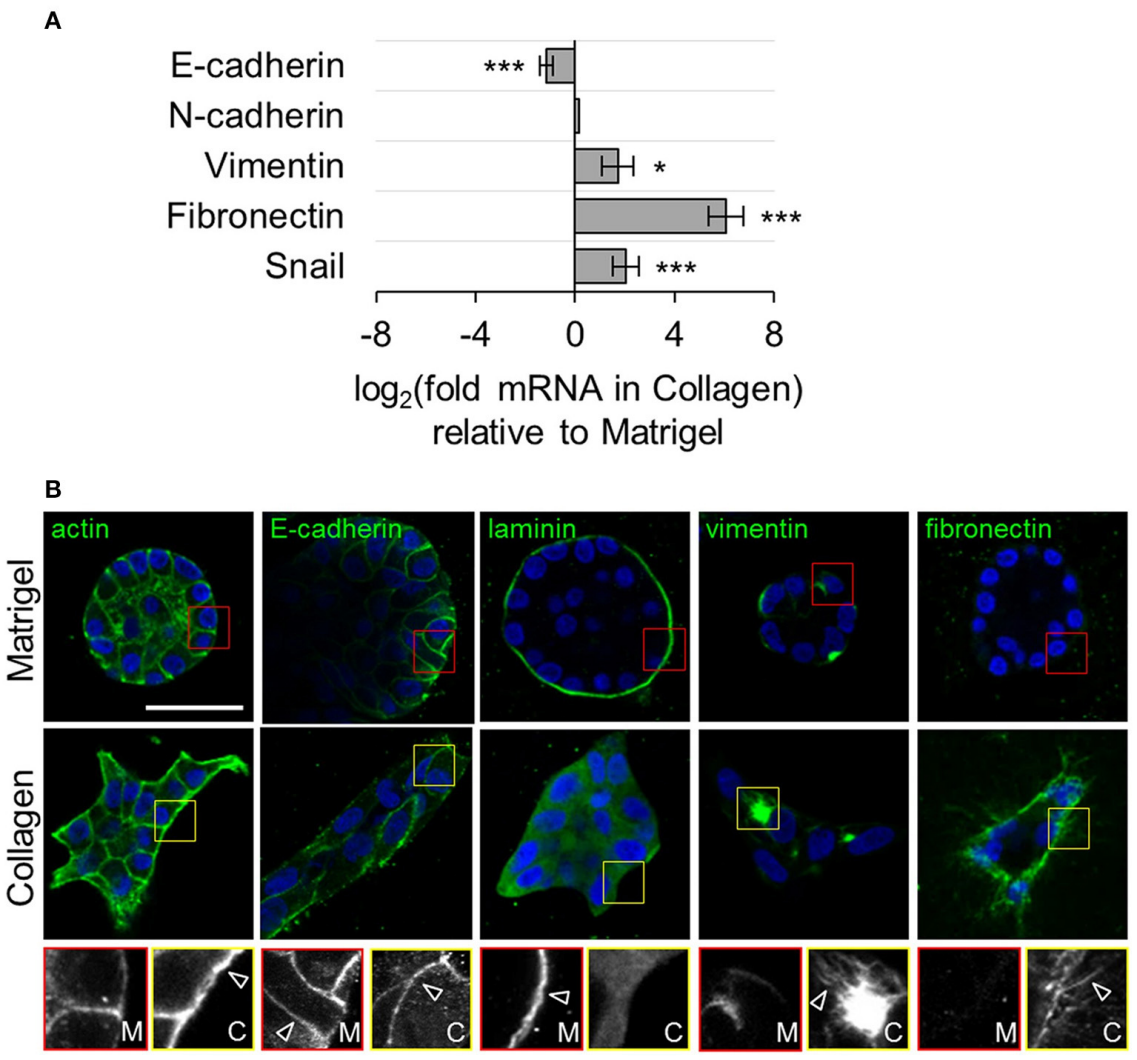
Benign epithelial MCF-10A have mesenchymal-like gene expression and invasive morphology when grown in a matrix that mimics tumor environments. Matrigel was used as a control that mimicked the properties of healthy breast tissue and collagen mimicked a cancerous scaffold. **(A)** E-cadherin was downregulated and mesenchymal markers vimentin, fibronectin, and snail were upregulated in collagen. *p = 0.01, ***p < 0.0001. **(B)** Staining of actin, E-cadherin, and mesenchymal markers with confocal imaging revealed normal acini structures in matrigel and protrusive and invasive structures in collagen. Regions of interest are in red for matrigel and yellow for collagen with arrows marking notable features. Scale bar is 50 μm. Reprinted with permission from Springer Nature (Carey et al., 2017).

Migration out of the premetastatic niche exposes tumor cells to new ECM mechanical environments which may affect cancer progression. Such environmental changes likely function through specific ECM ligand and integrin interaction. For example, abnormally high stiffness, as can be seen in the bone metastatic milieu, can regulate bone-destructive phenotype in MDA-MB-231 cells *via* regulating specific integrin subunit (Page et al., 2015). It was found that parathyroid hormone-related protein (PTHrP), a factor associated with tumor destruction of bone, and integrin β3 expression showed increases with increasing substrate rigidity. Taking advantage of this, injection of MDA-MB-231 cells with blocked β3 integrin did not show tumor-induced bone destruction, providing a potential therapeutic target. Further down this mechanosignaling pathway, Src, PI3K, Rac1, and rho-associated kinase (ROCK) inhibitors were tested for the regulation of general invasive epithelial phenotype (Carey et al., 2017). Inhibiting Src and ROCK promoted invasive protrusions, whereas inhibiting PI3K and Rac1 suppressed invasion. When testing the stiffening of the ECM, adding stiffer collagen caused benign MCF-10A to have an invasive phenotype and promoted cell dispersion. Unlike culture in Matrigel which promotes healthy epithelial formation, stiffer collagen matrix caused an invasive epithelial phenotype with activated mesenchymal genes and proteins including vimentin, fibronectin, and Snail (Figure 1) (Carey et al., 2017).

Even if tumor cells are removed, the remaining cells may have been primed with the mechanical memory effect. Benign epithelial MCF-10A and cancerous MCF-7 cells displayed the mechanical memory effect influencing later migration capability (Nasrollahi et al., 2017). Cells that were seeded on a stiff polyacrylamide substrate and later moved to a soft gel had faster migration, larger focal adhesions, and higher actomyosin expression compared to cells that were primed on a soft matrix. This mechanical memory was dependent on yes-associated protein (YAP), which was translocated and retained inside the nucleus when cultured on stiff substrates, and inhibiting YAP significantly suppressed the memory-dependent migration. This provides evidence that epithelial cells can have lasting effects from local tissue stiffening. The mechanical memory effect may also exist in cells associated with the epithelial cells, e.g., cancer-associated fibroblasts. Primary fibroblasts isolated from tumors exerted higher traction force and had higher migration than fibroblasts from non-cancerous sources (Alcoser et al., 2015). In 3D collagen matrix, fibroblasts from breast tumors migrated twice as far as fibroblasts from non-cancerous regions, suggesting that cancer must be studied as a combined system and studying only tumor cells may not provide a thorough picture.

As suggested above, recently, a particular interest in the memory effect of substrate stiffness includes YAP. The results with *in vitro* culture can be further supported with the tissue and *in vivo* mouse models. It was observed that initiation of metastatic colonization by organ-specific MDA-MB-231 (which is brain, bone, and lung metastatic) in capillaries was achieved *via* activated YAP (Er et al., 2018). Disseminated MDA-MB-231 cells used cell adhesion molecule L1 (L1CAM) to spread on capillaries, thus activating YAP *via* β3 integrin expression. The resultant replacement of local pericytes by cancer cells drove the early metastatic invasion. Similarly, cancer associated fibroblasts (CAFs) may require YAP mechanotransduction for formation and maintenance. In fibroblasts collected from various stages of cancer progression, feedforward regulation was found in which YAP regulates cytoskeletal tension and increasing this tension causes further elevation of YAP (Calvo et al., 2013). Inhibiting ROCK could prevent this feedforward loop, reversing the cancer-associated fibroblast phenotype. These mechanisms may not be limited to interactions between tumor cells and fibroblasts, but also have been noted in a two-way regulation in mesenchymal stem cell (MSC) differentiation to CAFs by factors from mammary cancer cells and CAFs in turn promoting cancer development. Specifically, soluble factors from cancer cells caused MSC differentiation to CAFs on stiff surfaces, but this CAF-forming mechanism was lacking when MSCs were seeded on soft substrates (Ishihara et al., 2017). It was suggested that MSC promotion of tumors is partially dependent on local tissue stiffness which acted *via* YAP and myosin light chain.

Development and maintenance of cancer stem cells may depend on the physical characteristics of the tumor microenvironment. Studies have screened substrate stiffness to determine the condition for the maintenance of cancer stem cell populations. It was observed that cancer stem cell marker expressions in MCF-7 and MDA-MB-231 were most prevalent at substrate stiffness of 5 kPa compared to non-optimal substrate stiffness of 2, 25, 50, and 70 kPa (Jabbari et al., 2015). Moreover, growing cells on an unphysiological stiffness of around 1 kPa, similar to tumor stiffness, caused tumor-like development compared with cells grown on soft native mammary tissue stiffness in the range of 150 Pa (Stowers et al., 2016). In the study, MCF-10A cells grown in soft hydrogel for 14 days formed healthy acini and exhibited quiescence. When the gels were stiffened after 14 days to mimic tumor stiffness, this caused cell proliferation, Ki-67 expression, and an invasion to the surrounding gels away from the acini. Examining mechanotransduction inhibitors evidenced that inhibiting PI3K/Rac1 pathway decreased the size and number of invasive acini. Similarly, Wei et al. (2015) showed Eph4Ras and MCF-10A established ductal acini and did not invade the basement membrane when grown on polyacrylamide gels that mimicked the natural 150 Pa stiffness of native tissue. However, when grown on 5.7 kPa gel that mimicked the stiffness of tumor environments, cells exhibited epithelial-mesenchymal transition (EMT) causing the invasion of the basement membrane. As a mechanism, the knockdown of TWIST1 could prevent the invasion of the basement membrane on stiff gels. TWIST1 was found to translocate from the cytoplasm to the nucleus on a rigid matrix for MCF-10A, MCF-10DCIS, Bt-549, and Eph4Ras cells, and blocking β1 integrin inhibited the invasion and prevented TWIST1 nuclear translocation.

Going beyond cytoskeletal tension and measures of invasion, the scaffold stiffness likely influences many other processes including microvesicle (MV) release and cell metabolism. Cancer cell derived MVs may contribute to remodeling of the surrounding environment, drive remodeling in the local premetastatic niche, and possibly primes distal sites for invasion. For example, MVs released from MDA-MB-231 cells activated fibroblast proliferation, contractility, and spreading in a matrix-stiffness dependent manner (Schwager et al., 2019). When MVs were applied to fibroblasts on substrates with the stiffness of healthy breast tissue, no changes were observed. However, when MVs were applied to fibroblasts on stiffer substrates mimicking tumorigenic breast tissue, MVs promoted spreading, cancer associated fibroblast-like phenotype, and contraction. In contrast, MVs from benign MCF-10A cells did not activate the cancer associated fibroblast behaviors on any substrates.

Besides substrate stiffness and related mechanical signaling to affect cancer cell behavior, exploring matrix composition and architecture may also be necessary. It was reported that cell energetics as measured by the ATP:ADP ratio depended on the matrix composition and the extent to which the matrix facilitated migration, e.g., ATP:ADP ratio in MDA-MB-231 was increased on cell migration impairing denser matrices, but decreased on migration facilitating matrices composed of aligned collagens (Zanotelli et al., 2018). The result is relevant for the tradeoff of leader and follower cells in that the coordinated relay-style tradeoff can be regulated by the energy state of the leader cells (Zhang et al., 2019).

For investigating breast cancer metastasis to soft tissues such as brain, soft matrices are particularly needed. Brain metastatic MDA-MB-231Br grown on hyaluronic hydrogels with a range of physiologically relevant, brain-mimicking stiffness displayed significant increases in spreading, adhesion, proliferation, and migration with increasing stiffness (Narkhede et al., 2018). Increased migration speed in particular depended on well-developed actin cytoskeleton on the stiff matrix. As a related governing mechanism, blocking focal adhesion kinase (FAK) on stiff hydrogels suppressed the cell adhesion, proliferation, and migration responses. Building on these, in a subsequent study, brain metastatic MDA-MB-231Br and BT-474Br3 cells were tested for dormancy on gels with varying stiffness (Narkhede et al., 2020). Brain metastatic breast cancer cells were dormant, as assessed by lower EdU and Ki67, when seeded on soft (0.4 kPa) hyaluronic acid hydrogel in comparison with the culture on stiffer (4.5 kPa) gel. FAK was again observed to mediate the stiffness-dormancy response and FAK inhibition increased the survival of dormant cells on stiff hydrogels. More model systems that recapitulate a variety of metastasis sites, both mechanically and chemically, are required to further study dormancy and ultimately prevent metastasis from occurring.

### Substrate Surface Patterns

**Figure 2:**
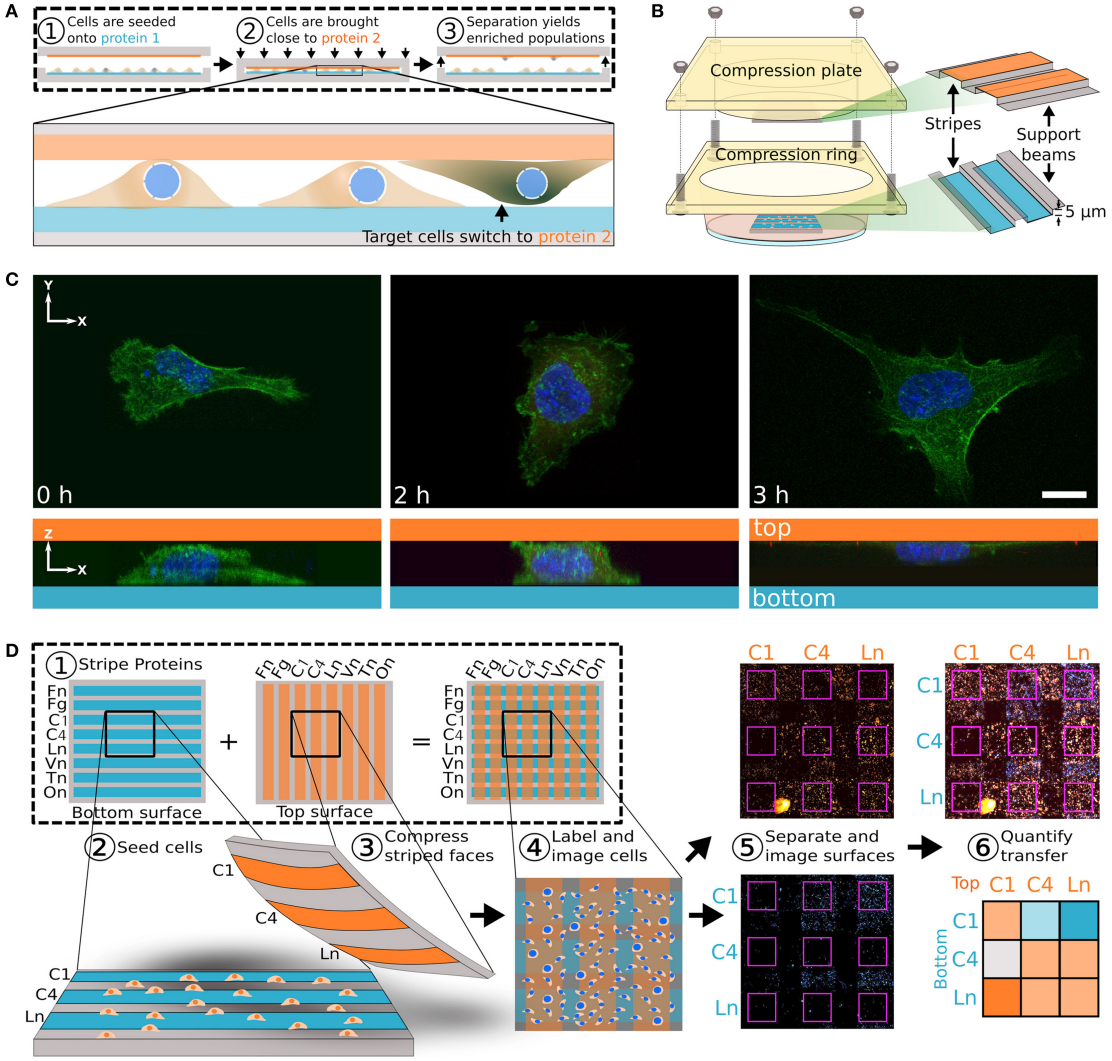
Transhesion assay characterizes cells based on adhesion molecule preference. **(A)** Schematic for transhesion method. Cells are seeded onto protein 1 and brought into close contact with protein 2. **(B)** This method is adapted to use a standard petri dish. **(C)** Cell imaging during transhesion demonstrates cell transfer from protein 1 (left) to protein 2 (right). **(D)** Eight orthogonal strips of proteins allows for testing of 64 protein combinations at once. At far right is a heat map with darker orange indicating preference for top surface and darker blue indicating preference for original bottom surface. Fn, fibronectin; Fg, fibrinogen; C1, collagen 1; C4, collagen 4; Ln, laminin; Vn, vitronectin; Tn, tenascin C; On, osteopontin. Reprinted with permission from Cell Press (Kittur et al., 2017).

Invasion and metastasis of cancer cells are likely dependent on abnormal adhesion to the surrounding environment. A better understanding of cell-ECM interaction is required as current prognostic methods do not completely predict metastasis. Patterning cells *via* controlling cell confinement within predetermined size, shape, and interconnectivity enables testing of cell morphology dependent fate decision, cytoskeletal tension, cell-cell interaction, etc. (Poudel et al., 2012). Also, when cells are seeded on anisotropic or isotropic topography patterns, cells display contact guided orientation to the anisotropic direction or functional behavior changes depending on the scale of the isotropic topography (Lim and Donahue, 2007). Cells may be characterized based on adhesion properties to surface patterned ECM proteins. Specifically, adhesion-based biomarkers in breast cancer cells can be identified by subjecting cells simultaneously to two ECM protein patterned surfaces and pulling the surfaces apart (Figure 2) (Kittur et al., 2017). It was observed that malignant MDA-MB-231 and the organ-tropic variants TGL/1833, TGL/4175, and TGL/Brm-2a (which are bone, lung, and brain metastatic, respectively) tend to transfer away from basement membrane proteins to collagen type I, while benign human mammary epithelial cells (hMECs) did not show such a transfer. If integrins α2 and β1 were inhibited, the transfer to the secondary ECM pattern was blocked. This adhesion profile method may provide crucial data for patient specific treatment depending on cancer cell-ECM interactions. Inspired by this, this section will discuss the control of breast cancer cells with two types of substrate patterning, the creation of topographic patterns in the form of geometric structures that cells may interact with and chemical patterning for selective culturing of cells within defined micropatterned regions. Also, examples using related techniques are included.

Differential response to topographic features is evident between healthy epithelial cells and malignant cancer cells. Malignant MCF-7 cells displayed significantly decreased vascular endothelial growth factor (VEGF) synthesis with lower proliferation and increased apoptosis when seeded on poly(lactic-co-glycolic acid) (PLGA) surfaces with 23 nm topographic features relative to cultures on smooth or 300 or 400 nm topographies (Zhang and Webster, 2012). On the other hand, benign epithelial cells had increased proliferation on the 23 nm features compared to the other surface conditions. This raises the potential of nanoscale topography for anticancer treatment. The anticancer property may be further enhanced with functional surface coating (Zhang and Webster, 2013). Coating the nanoscale features with alginate further decreased the cancer cell function whereas benign cells were unaffected. Beyond cell survival, topographic patterning of microscale features can influence cell migration and cytoskeletal composition. MDA-MB-231 cells had the greatest migration and cell speed on the arc pattern compared with grating pattern or flat control surface (Zhou et al., 2017). The uneven pattern of the arcs could create uneven vinculin focal adhesion protein expression within the cell, possibly causing the increased migration. Taking the topography-induced migration a step further, Chen et al. (2019) biased the direction of cell migration with sawtooth nanoscale ridges formed by multiphoton absorption polymerization. MDA-MB-231 preferentially moved up the sawtooth formations and the M4 metastatic variant of the MCF-10A cell line moved in the opposite direction. With these cases considered, topography could be a powerful tool for affecting breast cancer cell migration and interrogating the molecular motility pathways.

Micropatterning and related techniques can provide advanced methods for developing co-culture and more robust tumor models. In addition to sophisticated lithography-based patterning techniques, making PDMS mold for gradient microfluidic chips could be used to assess cancer cell migration (Zhao et al., 2018). In their study, when cells were patterned on collagen coated plates to measure the migration from the tumor regions into fibroblast regions, MDA-MB-231 had the greatest migration and MCF-7 were relatively stationary. Using defined patterns, cells from tumor regions could later be harvested for the gene expression analysis. Such microfluidic systems were also used to quantify cell migration as affected by chemotherapy drugs or other treatments (Wang et al., 2012). Moving to 3D culture, microfabrication using stereolithography could encapsulate MDA-MB-231, MCF-7, and MCF-10A in gelatin methacrylate to serve as a tumor model (Peela et al., 2016). Using time lapse imaging, cells escaping the 3D tumor-like encapsulation could be quantified and migration tracks compared, in which MDA-MB-231 showed pronounced invasion to the surrounding soft matrix. By adding endothelial cells to the 3D patterning models, the vascular interactions may also be investigated, for instance, breast cancer cells were patterned with endothelial fibroblasts in hyaluronic acid and fibronectin gels for the study of angiogenesis in the tumor model (Dickinson et al., 2012). The 3D printed nanocomposite matrix can also be used for breast cancer cell metastasis to the distal site such as bone (Zhu et al., 2016). In the study, to provide bone-mimicking niche, hydroxyapatite nanoparticles were suspended in a hydrogel. When grown with MSCs which can differentiate to bone-forming cells and deposit bone matrix, the tumor cells formed spheroids similar to those found when breast cancer metastasizes to bone. Interestingly, the cancerous cells in the biomimicking 3D environment displayed higher drug resistance compared to those grown in a 2D environment, indicating the need of further investigation of 3D bone-like nanocomposites for tumoroid studies.

**Figure 3:**
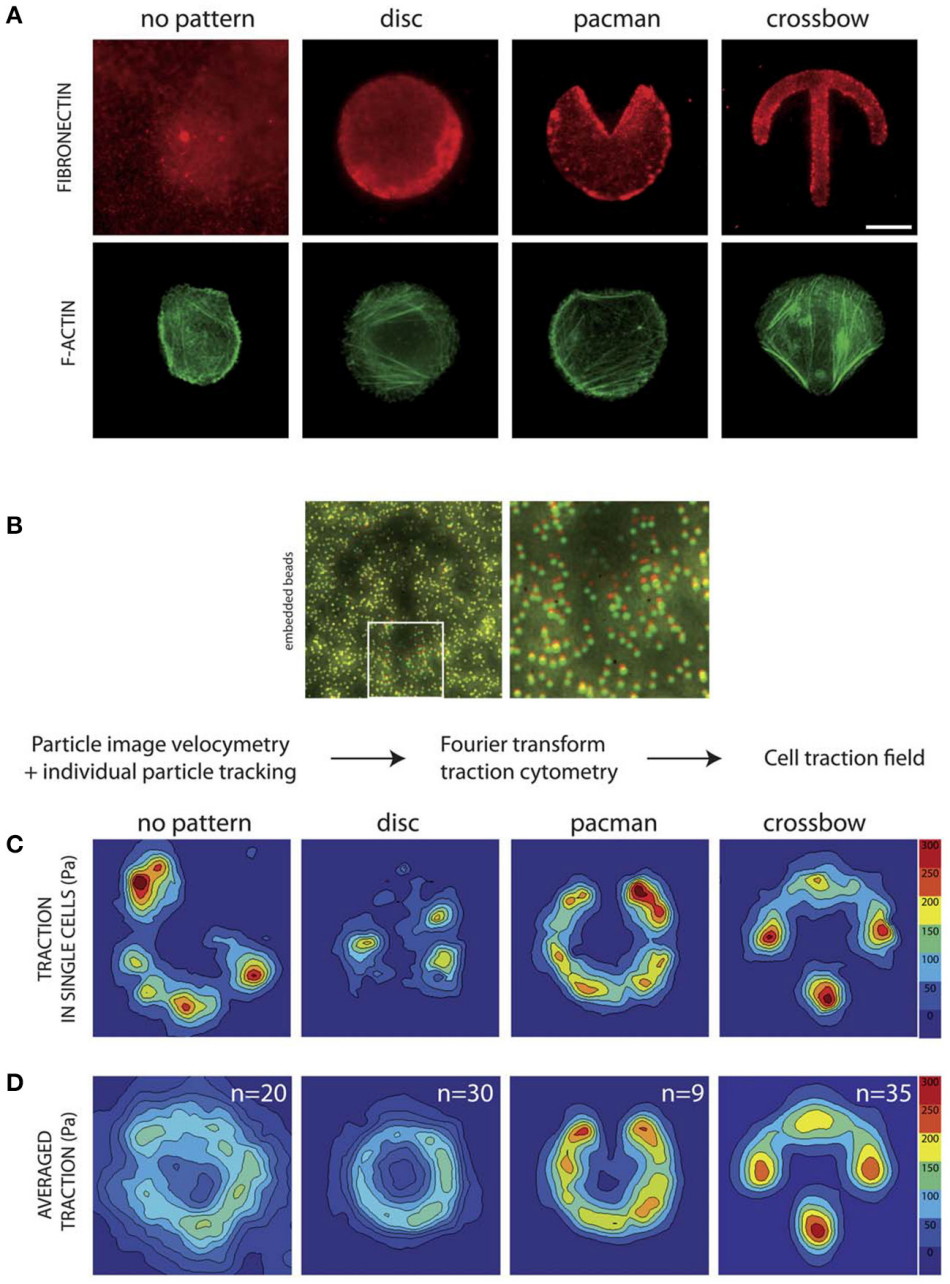
Patterning may be used to investigate cytoskeletal structures and cell traction force profile for mechanotyping. **(A)** Representative images with staining for F-actin and fibronectin reveal cytoskeletal organization. **(B)** Beads embedded in the substrate beneath the patterned surfaces can be used to determine displacement fields for traction measurements. Using this method the traction force field can be determined for **(C)** individual cells and **(D)** averaged for the pattern. This method provides high resolution data on cell traction forces which may be used to estimate invasive capabilities. Scale bar is 10 μm. Reprinted with permission from The Royal Society of Chemistry (Tseng et al., 2011).

Besides creating model systems, substrate patterning methods can also be used to interrogate physical properties of cells and distinguish between benign and malignant cells. For example, cell micropatterning was used to assess cytoskeletal stress and arrangement and infer cell state and malignant status (Figure 3) (Tseng et al., 2011). In their study, benign epithelial MCF-10A cells were patterned on fibronectin micropatterns having crossbow, disc, and pacman shapes, and traction force measurement was combined to reveal cell strains at each location on the pattern. It was observed all transformative tumor characteristics were not entirely involved with increased cell contractility, which result is potentially contradictory to the current general view. Since tumor cells often have modified contractile properties, such a system may be useful for assessing cancer cell progression. Similarly, the deformation of the nucleus may reveal the status of cancer cells. The nucleus is physically connected to cytoskeletons *via* the linker of nucleoskeleton and cytoskeleton (LINC) complex, e.g., nesprin and SUN, and may have a direct role in environment sensing in many cell types (Bouzid et al., 2019). To test the correlation of LINC and nucleus morphology with metastatic potential, topographically patterned micropillars were used to culture MCF-10A, MCF-7, and MDA-MB-231 cells (Matsumoto et al., 2015). Matching with the trend of highly metastatic cells having lower cytoskeletal tension, more metastatic cells displayed higher measures of nuclear deformation associated with significantly reduced LINC expression. The patterning technique has also been attempted to test cellular tension-related multinucleation which is a cancer-related adaptation. Cancer cell fusion is known to contribute to tumor development with fused cells having higher drug resistance. The metastatic MDA-MB-231 cell line formed multinucleated cells within confined patterns, but the typically stationary MCF-7 cell line did not (Zhu et al., 2019). Notably, multinucleated cells first formed on the edges of patterns in higher tension regions and later formed in the middle of patterned regions, revealing a potential strain-sensitive process for the multinucleation. Together, cell micropatterning could be useful for determining cytoskeletal tension and nuclear properties which regulate tumor development and metastasis.

Additionally, cell micropatterning has been utilized to examine dynamic cancer cell processes including migration. A two-state micropatterning with two large rectangular regions connected by a narrow bridge was developed to determine the heterogeneity in migration for established cancer cell lines (Brückner et al., 2020). The migration of MDA-MB-231 cells seeded on one side of the pattern and crossing the small bridge to the second region was recorded and assessed with statistical analysis to reveal potential subpopulations with distinct mechanical and migration capabilities. In a study by Tse et al. (2012), micropatterned cancer cells produced migration leader cells at sharp corners of the pattern in uncompressed controls. When adding the mechanical signal of compression to these patterns, the cells at the pattern boundary were likely to become leader cells. Again suspecting the role of cellular tension in this process, ROCK or MLCK inhibition blocked actomyosin remodeling and diminished the overall migration but did not stop the formation of leader cells.

Studying migration behavior in a patterned environment with physical constriction found the physical constraints to be an important factor in breast cancer migration speed. MCF-7 cells in narrow channels with high constriction migrated faster than those in wider channels, while benign cells (L929, HEK-293) did not migrate faster in confinement but migrated faster in less confined patterns (Yang et al., 2016). These changes were influenced by altered cell stiffness, e.g., cells in the patterns had elevated stiffness as measured by AFM compared to those growing freely without pattern. This system may thus reveal cell behavior relevant to metastasis in narrow capillaries or microchannels. In combination with the topography control of breast cancer cell migration (Zhou et al., 2017) described above, micropatterning and topographical features could provide tools for mechanotyping dynamic aspects of breast cancer cells such as migration and thus determining their metastatic potential.

### 2D vs. 3D Cultures

Although 2D models provide crucial data on cancer behavior, distinct oncogenic advantages are clear in even simple 3D cultures. We here highlight a few important implications of 2D and 3D cultures being used for breast cancer cell studies and based on that will further motivate the use of 3D culture systems. For a more detailed discussion of a variety of 3D models of breast cancer cells and tissues, see the review by Clegg et al. (2020) published in the journal of *Front Cell Dev Biol*.

**Figure 4:**
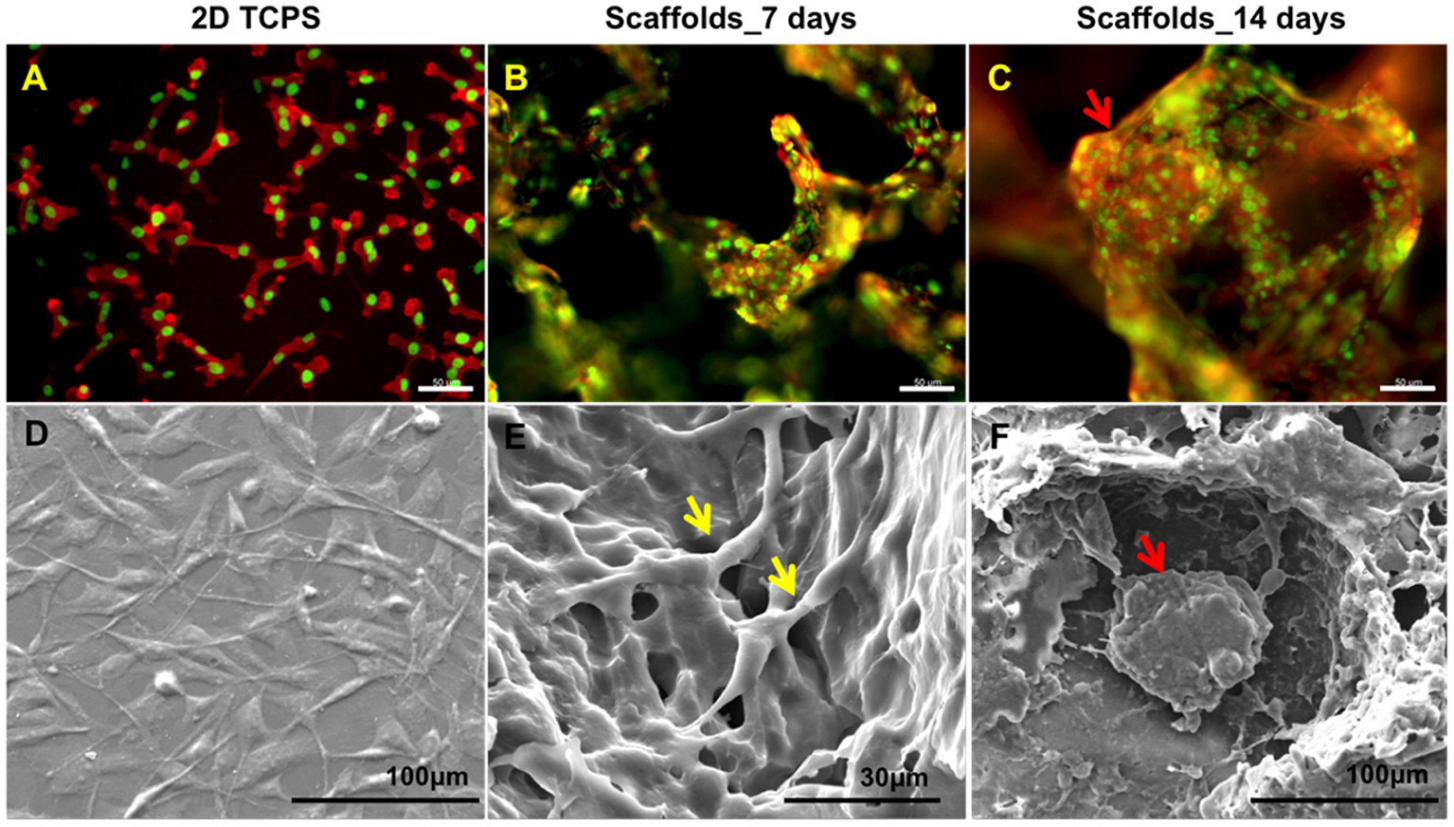
Growing cells in 3D scaffold that mimics breast tumor induced tumor spheroid formation. **(A)** Fluorescent imaging revealed that MDA-MB-231 on 2D tissue culture plastic (TCPS) failed to mimic the *in vivo* environment compared to scaffolds at **(B)** day 7 and **(C)** day 14. Staining for actin (red) and nucleus (green). Scale bars are 50 μm. **(D–F)** are the corresponding SEM images showing detailed cell interaction and tumor formation. Intercellular contacts are noted by yellow arrows and tumor spheroid are noted by red arrows. Reprinted with permission from American Chemical Society (Balachander et al., 2015).

Growing triple negative MDA-MB-231 in a 3D poly(ε-caprolactone) scaffold that mimics breast tumor induced notable gene activation associated with cancer invasion and motility compared to those grown on conventional 2D surfaces (Figure 4) (Balachander et al., 2015). This includes changes in invasive membrane structures (blebbing, lamellipodia) and genes related to cancer stemness (Notch1, Oct3), inflammation (NK-κB, TNF, IL8), and cell-cell and cell-matrix adhesion (integrin, MAPK, LINC, FAK, etc.). Moreover, preconditioning in a 3D tumor-like environment had lasting effects on cells as observed in the tumorigenicity mouse model, e.g., cells from 3D culture developed larger tumors in the mammary fat pad with local invasion and more metastasis to the lungs. Further understanding these processes could lead to better 3D scaffold designs for cancer detection and treatment. Further implementing realistic features to such models, the addition of a basement membrane and surrounding collagen ECM matrix into the 3D model allowed interrogation of the initiation of metastasis and basement membrane breaching (Guzman et al., 2017). In this setup, the basement membrane contained benign MCF-10A cells, while the oncogenically transformed MCF-10A-HRas invaded through the basement membrane endothelial layer and colonized the surrounding collagen matrix. In addition, 3D scaffolds designed to capture metastatic cells could decrease tumor burden at typical metastatic sites and alter the primary tumor milieu (Rao et al., 2016), e.g., poly(ε-caprolactone) scaffolds implanted in mice significantly reduced tumor progression in the liver and brain. Such 3D scaffolds may also serve as a diagnostic tool for monitoring the capture of metastatic cells, and cells captured in this manner may be further analyzed for patient-specific therapy. Adding another dimension of complexity, implanted scaffolds also modified the immune reaction in the primary tumor environment causing an invasion-suppressing profile (Aguado et al., 2018). Based on these data, further targeting 3D scaffold design for the control of oncogenesis and potential treatment and monitoring strategies will greatly benefit the field.

Developing 3D model systems and methods to study cancer metastasis will greatly enhance understanding of cancer disease progression, dormancy, and anti-metastatic drugs. To better mimic human bone environment as a breast cancer metastasis site, a tissue engineering approach was utilized to make nanoclay-based bone model (Kar et al., 2019). MSCs grown in a nanoclay matrix for a month could develop a bone-like tissue, and the construct was then seeded with MCF-7 and MDA-MB-231. This enabled investigations of 3D tumor spheroid formation, cell-cell and cell-matrix interactions, and metastasis potential. Taking the perspective of tumors as interconnected organ systems, further work is clearly required to develop model tumor systems that provide realistic cell-cell interactions and recapitulate the response of multiple cell types to mechanical signals (Bregenzer et al., 2019). For example, one study recapitulated 3D blood vessels with a parallel epithelial-lined lumen to investigate the epithelial-endothelial migration in breast cancer (Devadas et al., 2019). This is of significance since the endothelial-epithelial interactions are not well-understood yet for 3D environments. Moreover, the 3D environment is particularly important for endothelial culture since 2D monolayers of endothelial cells may behave differently than 3D lumens as measured by pro-angiogenic secretory factors (Jiménez-Torres et al., 2019). In comparison, attempting this work in less biomimetic 2D environments has relied on transwell inserts which lack the physiological 3D lumen structures and fluid flow stimulus.

In addition to modifying cellular crosstalk, the mechanical environment further affects cellular processes such as chromosome segregation. It was observed that fewer segregation defects occur in 3D epithelia culture compared to the segregation in 2D culture conditions (Knouse et al., 2018). In another study exploring the interaction of Ras oncogene with environmental stiffness in benign MCF-10A, Ras promoted cell rounding during mitosis even under confinement conditions, which then prevented chromosome segregation errors (Matthews et al., 2020). The forces generated by cells in this case were proposed to be different in 2D vs. 3D, with cell division in 2D resulting in selective loss of cell adhesion to the substrate whereas cell division in 3D may generate forces in all directions.

Taking these microenvironment-dependent cell changes one step further, the epigenetic profile could be sensitive to dimensional and stiffness effects. Stiff 2D culture environments may cause loss of tissue function due to changes in chromatin. In a 3D culture model, stiff ECM induced tumorigenic features in mammary epithelial cells with increased lamina-associated chromatin with more accessible chromatin; a soft ECM in the range of the natural *in vivo* environment caused *in vivo* like chromatin profiles (Stowers et al., 2019). Tying in topographic patterning, simply imprinting cell membrane shapes in culture surfaces drastically altered apoptosis and susceptibility to breast cancer drug treatment (Shahriyari et al., 2020). Although 2D cultures have been used a lot for breast cancer work, all of these data from 3D culture setups demonstrate that the culture dimensionality together with the mechanical microenvironment likely affect a variety of cancer cellular processes. Further motivating the move to 3D culture environments, proper functioning of cellular processes in a more physiological way can be achieved which are inhibited in 2D in many cases. Some representative concepts/findings of the microenvironmental (stiffness, patterning, 2D vs. 3D) control of breast cancer cells are shown in Table 1.

## Effects of Mechanical Loading Milieus

### Mechanical Stretch

Among various mechanical loading situations to which breast cancer cells could be exposed *in vivo*, mechanical stretch has been relatively less investigated. Recently, response of breast cancer cells to mechanical stretching was suggested as a marker to distinguish between cancerous and benign cells (Yadav et al., 2019). Under cyclic stretching of 1.4% strain at 0.01 Hz frequency, cancerous MDA-MB-231 cells displayed in the initial stretching cycle decreased roundness, increased cell length, and actin cytoskeletal rearrangement perpendicular to the axis of stretching; in the later stretch cycles they showed apoptosis potentially due to increased cellular rigidity. These changes by stretch were specific only for MDA-MB-231 but not for non-cancerous cells, indicating that mechanical stretch can be utilized for identifying their metastatic potential.

Cytoskeletal integrity and rearrangement are crucial for cancer cell adhesion and invasion processes. It was found that stretched MDA-MB-231 cancerous cells showed many fold increase in invadopodia, the actin-rich protrusion of the plasma membrane associated with the cancer invasiveness (Ansaryan et al., 2019). Further, the activation of aerobic glycolysis and altered membrane voltage, both indicative of cancer progression, were observed in all breast cancer cell lines tested under stretch, indicating that stretch cue may significantly influence even benign epithelial cells to a cancer-like phenotype.

**Figure 5:**
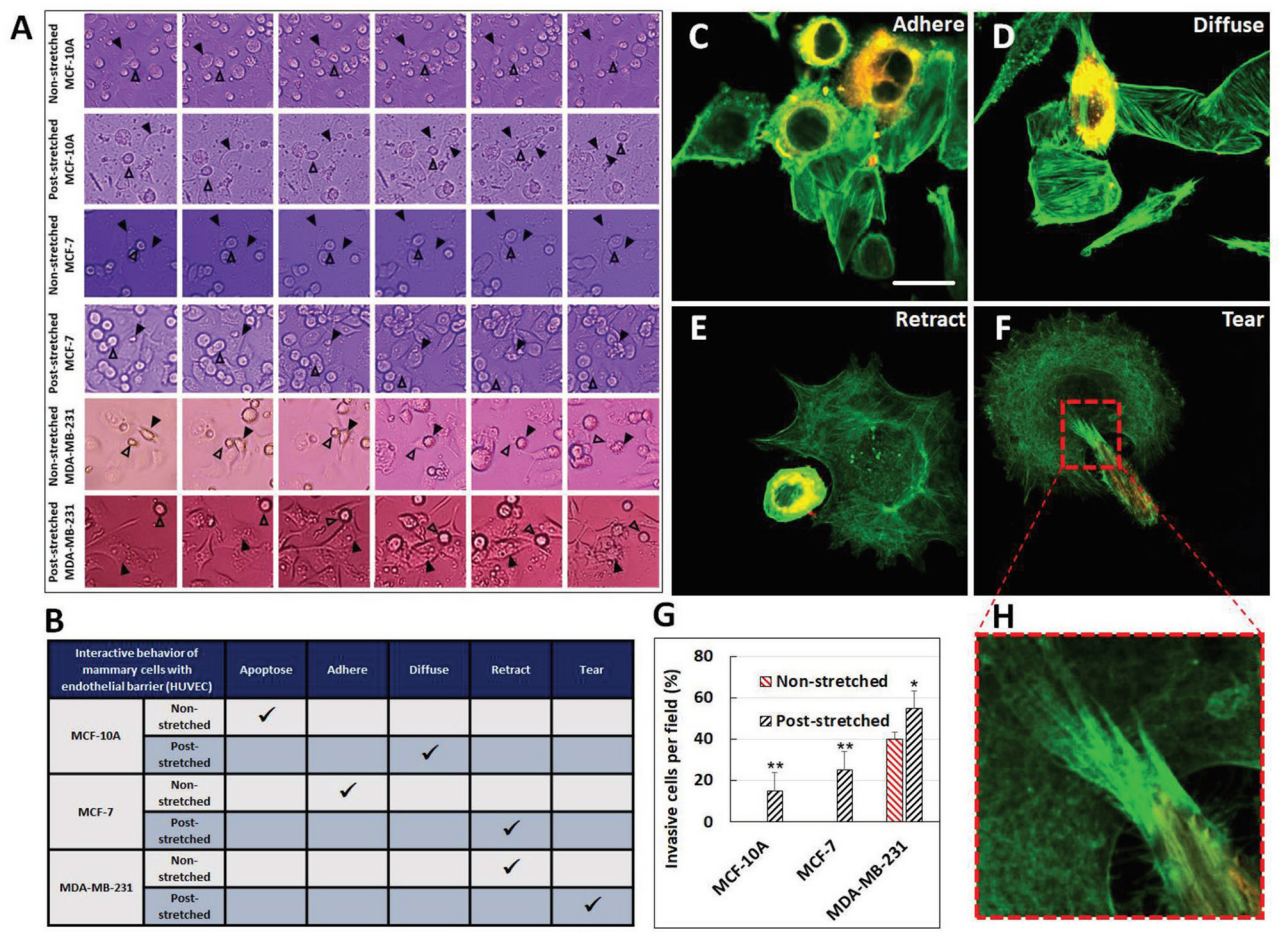
Tensile stretch caused escalation of invasiveness for benign MCF-10A and malignant MCF-7 and MDA-MB-231. **(A)** Timelapse imaging was used to track cell interaction behaviors over time. Behaviors of interest are marked with arrows. **(B)** This table summarizes the behavior of the cell lines in the static unstretched group and after stretch. Representative images of **(C)** adherence, **(D)** diffusion, **(E)** retraction, and **(F)** tearing invasive behaviors. Actin is stained in green for breast and endothelial cells, cancer cells also stained with Dil. Scale bar is 25 μm. **(G)** The number of invasive cells per field greatly increased with stretch. *p < 0.05, **p < 0.01. **(H)** Dense invadopodia tore the endothelial cells. Reprinted with permission from Wiley (Ansaryan et al., 2019).

Tensile stretching may activate motility pathways in multiple subtypes of breast cancer. When MCF-7, MDA-MB-231, and 4T1.2 cells were subjected to uniaxial cyclic strain for 48 h at 10% and 0.3 Hz or to a constant 10% static stretch, both oscillatory and static stretches increased cell proliferation and subsequent transmigration for all three breast cancer cell lines compared with unstretched control (Wang et al., 2020). Typically, MCF-7 and 4T1.2 had substantially larger transmigration in the oscillatory stretch condition. The stretch alteration of cancer cell motility can further be assessed with the invasion assay on endothelial layers, which is utilized to classify breast cancer cell response into distinct behavior categories: apoptosis, adherence to endothelial layer, diffusion through the layer, retraction of endothelial cells, and tearing of the endothelial cells (in the order of increasing invasiveness). In assays evaluating the effect of mechanical stretch on such invasion test (Figure 5) (Ansaryan et al., 2019), the most invasive line tested, MDA-MB-231, caused retraction in the control condition but physically damaged the endothelial cells by tearing the cell membranes in the stretched group. Also, benign MCF-10A, which underwent apoptosis in the static control condition, could diffuse through the endothelial layer after stretch. Similarly, non-invasive malignant MCF-7 adhered to the endothelial layer in the static control but caused retraction of the layer after stretch. Thus, all three cell lines, either benign or cancerous, displayed increased invasiveness when exposed to stretch. The invasion and anticancer drug resistance of MCF-10A, MCF-7, and MDA-MB-231 were also elevated in response to static stretch at 15% for 12 h.

This stretch-induced invasive phenotype persists for long periods even when injected into a mouse model. Cyclic stretch conditioned 4T1.2 cells transferred to BALB/c mice created significantly larger tumors than unstretched cells (Wang et al., 2020). The stretch conditioned cells also modified the local immune environment *via* exosome signaling which suppressed anti-tumorigenic immune response. Stretch conditioned cells had significant lasting immune modulation and tumor growth after 10 days, demonstrating that stretch conditioning had lasting effects which persisted when transferred *in vivo*.

To date, mechanical stretch studies on breast cancer have mostly focused on breast cancer progression. As another perspective, Berrueta et al. (2016) demonstrated that local stretching of tissue *in vivo* can reduce inflammation, fibrosis, and tumor volume. Mice implanted with breast cancer were subjected to 10 min of stretching from forelimb to tail daily, and this resulted in 52% smaller tumor volume compared with unstretched control mice. Some evidence of restoration of T-cell adaptive immunity against the tumor was also found with more abundant lymphocytes in the stretch group. Cytokine mediators of inflammation and cytotoxic immunity were upregulated in the stretched group, revealing a potential mechanism for the stretch-based tumor regulation. This study provides further evidence that factors in the tumor environment and surrounding cells react to mechanical signals in varied and nuanced ways, which may then be utilized in cancer treatments (Berrueta et al., 2018). Complex interventions such as stretching of *in vivo* tissue and model tissues should be continued to better understand and then resolve the mechanisms of cancer suppression and possible strain-based interventions.

### Compression

The nuanced effects of solid stress on tumor cell progression are poorly understood. Solid stress results from tumors growing in confined tissue environments: the cells increasing in number within the tumor experience compression and the surrounding tissue is remodeled with changes in blood and lymph vessels (Stylianopoulos et al., 2013). Pathological remodeling is also found in the ECM, as is related to ECM stiffening *via* collagen densification in compressive environments. This in turn affects viscoelastic relaxation, plastic deformation, and interstitial flows in the compressive tumor microenvironment (Ferruzzi et al., 2019). As tumors develop, its interior becomes hypo-vascular and continued cell proliferation causes vascular compression in the periphery. This tends to reduce the efficiency of drug delivery, thus the strategies to alleviate internal tumor stress and reduce cell proliferation may restore efficient drug delivery to the tumor interior (Mpekris et al., 2015). Stress resultant changes in hydrostatic pressure, interstitial flow, and chemical environment all interact to drive or inhibit the cancer progression. In a 3D MDA-MB-231 hydrostatic model (Tien et al., 2012), pressure on one side of an aggregate inhibited tumor outgrowth from the opposite side. When no pressure gradient was applied, the cells invaded the surrounding collagen matrix, extending tens of micrometers from the original tumoroid. The pressure caused interstitial flow in the aggregate which altered the local chemical microenvironment and resulted in inhibited cell outgrowth. This in particular underlines how mechanical stress signals, the local ECM environment, and chemical factors can be interconnected in tumor outgrowth.

**Figure 6:**
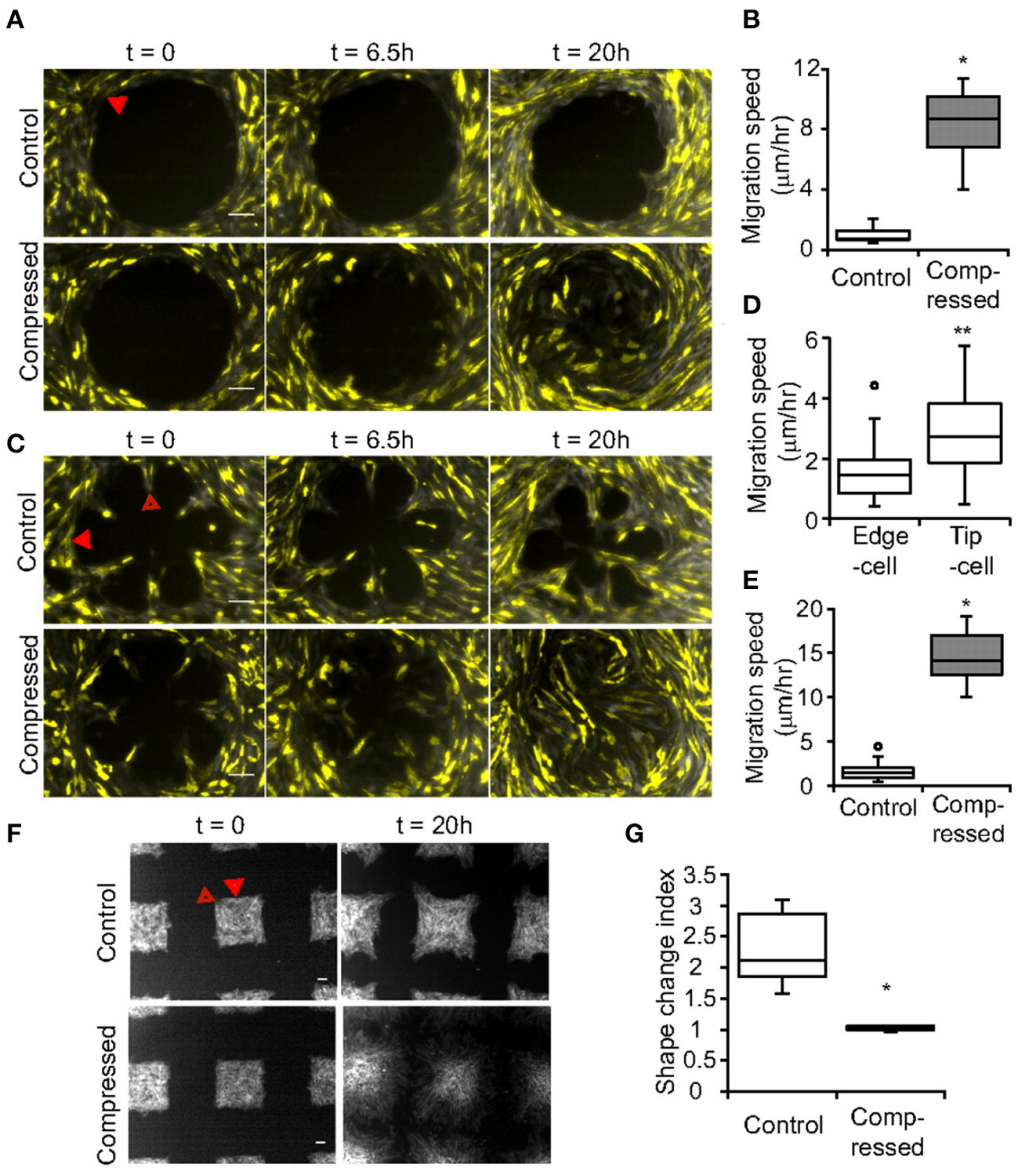
Compression caused enhanced invasion properties and formed more leader cells. In uncompressed controls, the pattern shape **(A)** circle, **(C)** rosette, **(F)** and square controlled leader cell formation. Scale bar is 100 μm. Compression greatly increased leader cell formation on the patterns with increased **(B)** speed in the circular pattern. **(D)** Migration speed of tip cells is greater than edge cells. **(E)** Migration speed is greater for compressed cells. **(G)** The square pattern shape change can be assessed with the shape index which significantly changed under compression. *p < 0.005, **p < 0.05. Solid arrow marks edge cells and hollow arrow marks tip cells. Reprinted with permission from National Academy of Sciences (Tse et al., 2012).

Compressive stress can alter cell adhesion to increase adhesive properties and promote invasive phenotypes. For example, in a test using 5.8 mmHg compressive pressure, compressed cancer cells exhibited actin and microtubule realignment with the cell sheets producing distinct leader cells at the periphery to guide the migration activity, and thus displayed persistent and directed migration motion compared with non-compressed control (Figure 6) (Tse et al., 2012). Such changes were only observed for invasive 67NR cells but not for benign epithelial MCF-10A or non-invasive MCF-7. In another study, MDA-MB-468 compressed by 15 mmHg pressure increased adhesion by 25% (Downey et al., 2006). Also, compression of MDA-MB-231 at 50% strain (0.05–0.25 kPa) for 3 h in agarose gels increased cell-ECM adhesion and migration while downregulating genes involved in ECM degradation (Demou, 2010). As a potential mechanism, compression of MDA-MB-231 was shown to induce the overexpression of integrin genes with increased expression of PTEN that is found at the leading edge of cell migration. In addition, platelet endothelial cell adhesion molecule-1 (PECAM-1) was downregulated and CD44, typically associated with the central nervous system, was significantly upregulated, revealing potential priming for specific metastasis sites such as brain. Combined, compression could transition tumor cells to a more invasive phenotype as measured by migration and gene expression with potential priming for specific distal metastasis sites.

Besides compression from increased tumor solid stress, invasion through ECM, intravasation, and extravasation also induce unique compressive strains *via* constrictions on migrating cells. In a study testing MCF-10A, MDA-MB-436, and MDA-MB-231 cell migration through narrow constriction using microfluidics and combined chemotaxing with epidermal growth factor (EGF) gradient, counterintuitively, it was evidenced even benign MCF-10A cells may have a natural propensity to migrate through constrictions (Ficorella et al., 2019). The mesenchymal-like MDA-MB-231 used blebs to pass through the constriction, whereas the MCF-10A primarily used lamellipodia with some blebbing. The less aggressive metastatic MDA-MB-468 did not adapt or optimize for passage through the constriction, resulting in low migration through the passage. These results may need to be interpreted considering the dimensionality and mechanical properties of the local environment. Mesenchymal migration strategies have been observed in 2D migration, while in 3D environments with constrained channels the cytoskeletal adaptation may reduce the dependence on focal adhesions. It was observed that MDA-MB-231 migration was reduced by β1 integrin inhibition on 2D substrate but not affected in 3D (Balzer et al., 2012). The directed migration in 3D confined environments relied more on microtubule polymerization but less on actin polymerization as is dominant in 2D. Understanding migration strategies in compressive constriction situations may be key to developing anti-metastatic treatments to block invasion through the ECM and intravasation.

How compression contributes to cancer cell survival and progression is largely unknown. Compression may contribute to tumor cell survival in the hypoxic conditions by activating glycolysis genes and adapting cell metabolism and microRNA (miRNA). It was observed that metabolic, EMT-related, and angiogenesis genes were all upregulated in compressed patient-derived cancer-associated fibroblasts compared to the static control (Kim et al., 2019). *In vitro* compression models also found upregulated glycolysis genes in MDA-MB-231 and migration-related genes in SK-BR-3 cells. In a study using a 3D agarose scaffold-alginate bead model, compression enhanced tumor phenotype in MDA-MB-231 and BT-474 cells and VEGF expression in the breast cancer cells and associated fibroblasts (Kim et al., 2017). This may act through the compression regulation of miR-9 which can regulate lamin and integrin and is known to be downregulated in advanced breast cancer. The expression of miR-9 was decreased in MDA-MB-231 and BT-474 breast cancer cells and cancer-associated fibroblasts but not in low metastatic MCF-7 or SK-BR-3, highlighting potential differences in miRNA among breast cancer subtypes. In another study, MDA-MB-231 cells had the largest response to compression which downregulated miRNAs associated with tumor suppression genes such as apoptosis, adhesion, and cycle arrest, whereas MCF-7 and BT-474 cells had lower response (Kim et al., 2016). Such interrogation may reveal miRNA targets for disrupting breast cancer and cancer associated fibroblast communication *via* paracrine signaling. Together, compression could regulate miRNA expression critical for tumor development and suppression, and therapeutics based on inhibition of tumor miRNA or enhancement of suppressive miRNA would provide targeted cancer treatments.

Inducing necrosis over apoptosis in cancer cells encourages immune cell recruitment. In addition to above-mentioned effects, compression was also tested for tumor cell necrosis. It was observed that necrosis was induced in BT-474 and MDA-MB-231 by compression in a force and time-dependent manner (Takao et al., 2019). Dynamic compression in the range of about 10 kPa could induce cell death dominated by necrosis; dynamic compression to cause this effect was with less magnitude than that required in static compression.

**Figure 7:**
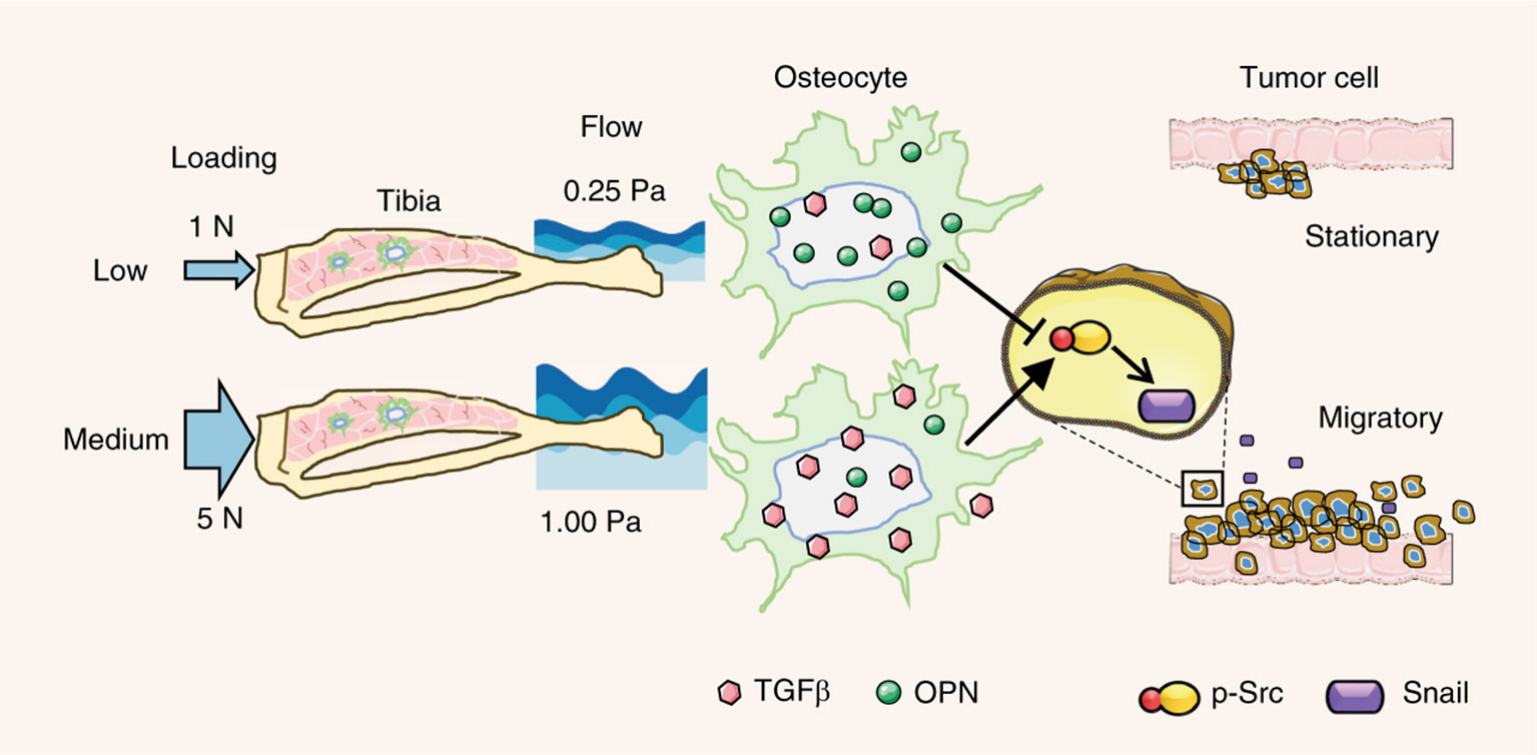
Loading magnitude regulates tumor destruction of bone *via* osteocyte signaling. Low levels of compressive loading of bone *in vivo* and of osteocyte shear *in vitro* caused suppression of cancer migration. Higher compressive loading of bone and shear of osteocytes caused activation of breast cancer migration by upregulating Src and snail. Adapted with permission from Springer Nature (Fan et al., 2020).

Similar to the initial tumor progression, the mechanical forces in distal metastasis sites can also guide cancer cell behavior. At the primary tumor site, cancer-associated fibroblasts contribute to tumor response and release cytokines upon stimulation. Fibroblasts interact with both the chemical and mechanical environments, providing additional mechanical cues and filtering of mechanical signals to the tumor (Bregenzer et al., 2019). In distal metastasis sites, this role is filled by other cells which interact with the metastasized cells. In one study, compressive loading of the bone in the mice that received intratibial MDA-MB-231 injection inhibited the tumor growth and prevented osteolysis associated with tumors (Lynch et al., 2013). This effect may act through the decrease of Runx2 in the loading group which is expressed by tumor cells and has an effect in both osteoclast and osteoblast regulation. In the absence of loading, the tumors continued to grow and osteolytic activity was increased producing a completely degraded tibia. Regulation of tumor development by compression may depend on the loading magnitude. For the tibia loading applied to mice injected with EO771 and 4T1.2 tumor cells, loading with 1 N reduced or prevented bone destruction from tumor activity, while 5 N induced osteolysis with significant bone loss and microcracks (Figure 7) (Fan et al., 2020). In parallel, if osteocyte-derived conditioned medium and fluid flow-treated medium were given to breast cancer cells at 0.25 or 1 Pa shear stress, both media under lower shear stress resulted in mesenchymal-to-epithelial transition (MET) while flow-conditioned medium at high shear triggered epithelial-to-mesenchymal transition (EMT).

### Fluid Shear

**Figure 8:**
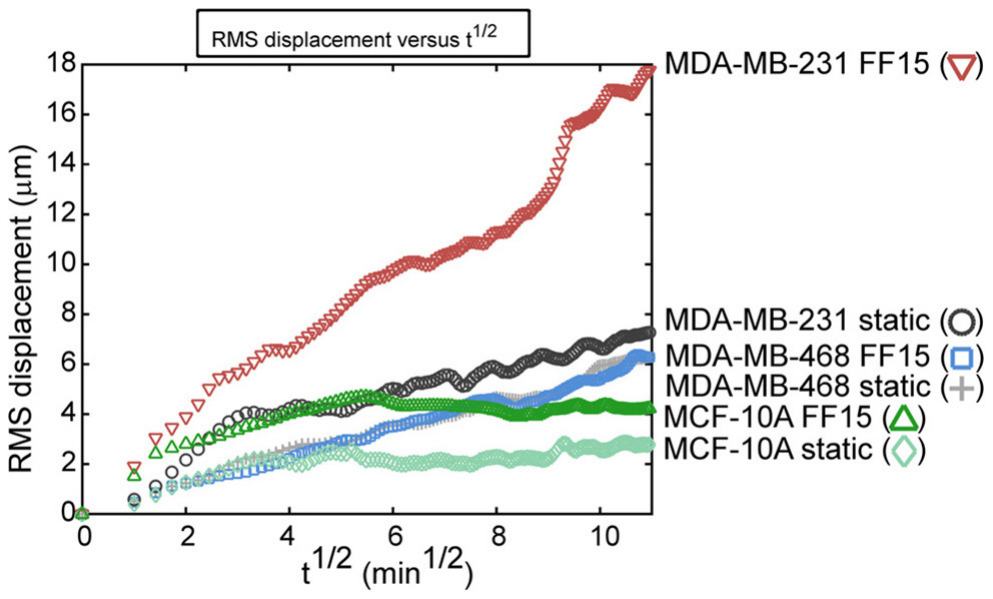
Group migration trends as assessed by root mean square (RMS) displacement reveal that intrinsic metastatic potential and fluid shear activation combine to increase breast cancer cell migration. Benign MCF-10A cells had low migration capabilities in both the 15 dyne/cm^2^ fluid flow (FF15) and static group. The less aggressive MDA-MB-468 cell line had almost no effect from fluid shear stimulation. Contrasting this, the aggressive MDA-MB-231 had higher migration in the static group and the migration capability was greatly increased by fluid shear stimulation. Reprinted with permission from American Society of Mechanical Engineers (Riehl et al., 2020).

Study of cell migration directed by fluid flow-induced shear is of significance for understanding how cells migrate out of tumors with the interstitial flow environment and for understanding how cells respond in active distal migration environments in the vascular, lymph, and metastatic sites. The direction of tumor cell migration is dependent on environmental factors including dimensionality, matrix material, cell density, flow velocity, and cell receptor activity, and MDA-MB-231 cells showed heterogeneous responses to microfluidic flows depending on the environmental parameters (Polacheck et al., 2011). In another study, fluid shear as a whole increased breast cancer cell motility in a 3D environment but again did so in a heterogeneous manner, so that simple averages of cell behavior might not reveal an accurate picture of migration (Haessler et al., 2012). Targeting higher shear flows as may be found in vessels, our group demonstrated that cells with higher metastatic potential display greater sensitivity in migration to fluid shear (as assessed by root mean square, RMS, displacement of all participating cell migrations as a holistic measure, Figure 8) (Riehl et al., 2020). With a parallel plate flow chamber producing 15 dyne/cm^2^ shear stress, we observed that flow caused highly metastatic MDA-MB-231 cell migration along the flow direction with higher displacement, greater speed, and less pausing. In contrast, less metastatic MDA-MB-468 was less responsive to flow and benign MCF-10A had the lowest migration potential under shear. Combined, these studies highlight how cells may have heterogeneous responses in migration under flow, which requires investigation in a range of flow environments simulating tumor or distal metastasis sites and for various breast cancer cell types.

In terms of potential mechanistic sensors to regulate migration under flow, for MDA-MB-231, interstitial flow-induced directional migration was blocked when CCR7 was inhibited (Polacheck et al., 2011). Also, FAK was activated specifically in cells that migrated against the flow direction and blocking Src kinase reduced migration against the flow. In another study targeting interstitial flow as present in tumors, it was found cells were polarized for migration through β1 integrin (Polacheck et al., 2014). Cell adaptation *via* paxillin focal adhesion anchor protein also had a key role in this process, producing biased migration in the flow direction, e.g., when paxillin was inhibited, MDA-MB-231 cells no longer migrated against the flow and instead moved with the flow. The details of integrin and related focal adhesion protein regulation under flow are still undetermined. It was recently proposed as part of the remodeling and adaptation response, integrin and focal adhesion components are trafficked by endocytosis (Tang et al., 2020). Fluid shear stress activated focal adhesion turnover in MDA-MB-231 by endocytosis, and β1 integrin was internalized and recycled back onto the cell surface during migration. This process was observed to be increased by shear flow stimulation.

Microfluidic platforms can provide an easily customizable solution for simulating tumor environments, for example, the microfluidic parallel lumen device targeting endothelial and epithelial co-culture as described above (Devadas et al., 2019). Devices may further be customized for each segment of the metastatic process. In a study by Chen et al. (2013), a 3D microvascular network was grown in a microfluidic platform to study cancer cell extravasation across endothelial cells and out of the lumen under shear stresses of 0.012–0.48 Pa relevant to those in venular microvessels. The transmigration rates were correlated with known metastatic potential with MDA-MB-231 having a rate of 13% and MCF-10A with only 5%. Revealing the mechanical aspect of transmigration, cells that were mechanically trapped or constricted had a transmigration rate of 48% compared to 10% for non-constricted cells. Understanding how flow in the blood, cell-cell interactions, and constrictions work together to influence the metastatic cascade could reveal new treatment targets. Cognart et al. (2020) tested with microfluidics epithelial-like and mesenchymal-like cells in their responses to the constriction in a flow. Constrictions in the flow could reveal the viscoelastic behavior of the cells tested: MDA-MB-231 demonstrated more plastic behavior evident in faster times for passing through subsequent constrictions after the first constriction, while this memory was less pronounced in epithelial-like SK-BR-3 cells. This work demonstrated changes in cancer cells generated by the mechanical stimuli that arise from circulatory conditions including constriction.

**Figure 9:**
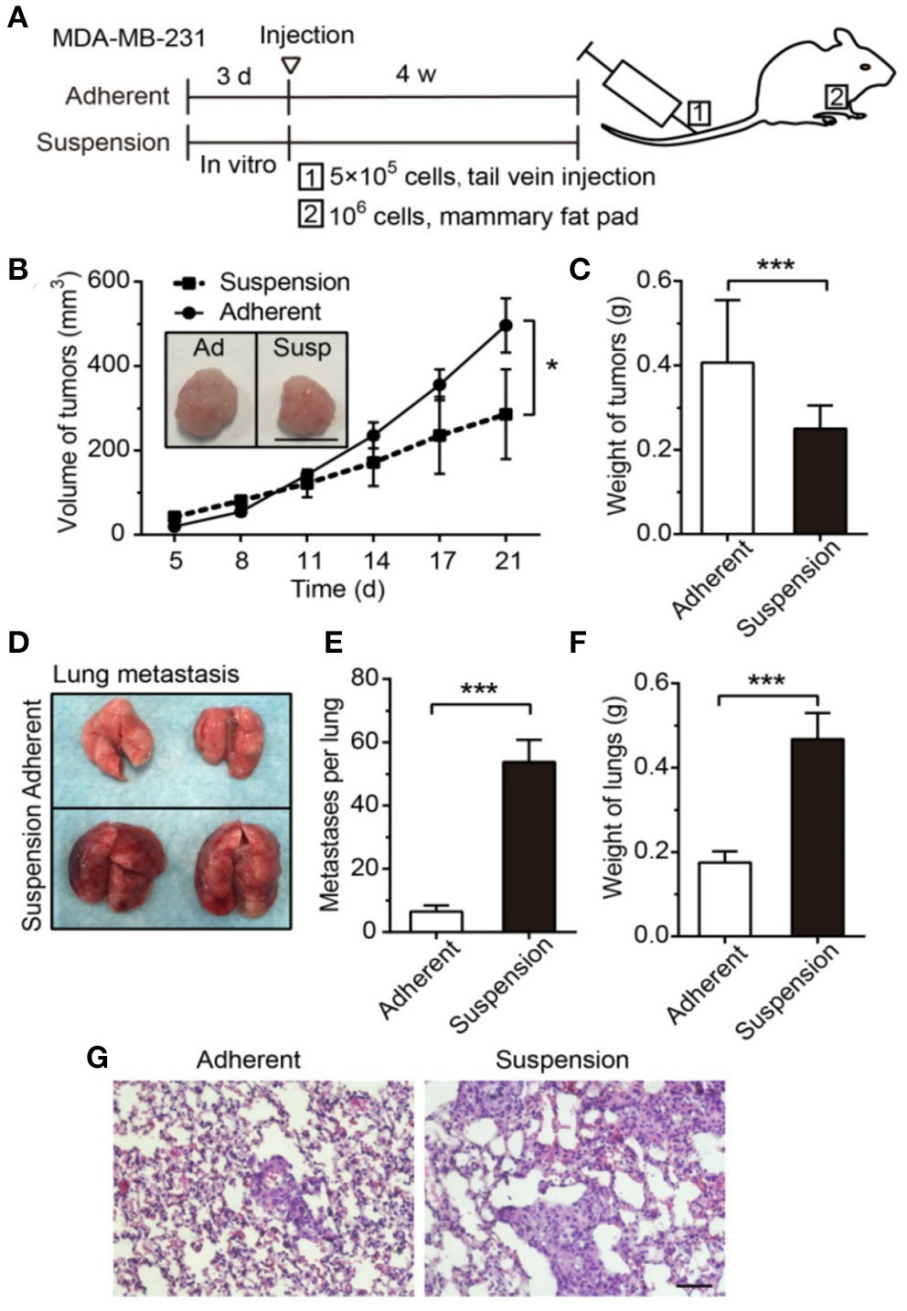
Suspension of cancer cells, as may be experienced in vascular migration, decreased volume of primary tumor but greatly enhanced lung metastasis. **(A)** The experimental timeline for 1: metastasis study for cell injected in the tail vein and 2: primary tumor from cells injected to the mammary fat pad. Suspension decreased the primary tumor **(B)** volume and **(C)** mass. Scale bar is 1 cm. **(D)** Representative lung images after 28 days reveal that suspension of cancer cells greatly increased metastasis as evident in the **(E)** number of metastasis and **(F)** mass. **(G)** Larger regions of metastasized cells in the suspension group is evident in H-E staining of the lungs. Scale bar is 50 μm. *p < 0.05, ***p < 0.001 Reprinted with permission from Ivyspring International Publisher (Zhang et al., 2018).

Flow causes a multitude of changes in cancer cell survival closely related to EMT including the development of stem cell-like properties. Although considered to be distinct, both EMT and stemness equip cells to migrate and survive and may combine to create migrating cancer stem cells. The flow shear may promote the EMT process and render cancer cells to be more aggressive by activating embryonic-like stem properties through the deactivation of extracellular signal-regulated kinase (ERK) and GSK3β (Choi et al., 2019). In a 3D flow bioreactor, shear stress significantly increased the area of MDA-MB-231, MDA-MB-468, and MCF-7 cells while decreasing roundness, which may be associated with higher propensity to metastasize (Novak et al., 2019). Tumor cells in the blood vessels, or circulating tumor cells (CTCs), are in a suspension state exposed to blood flows. It was found that cell suspension significantly increased the adhesion ability of MDA-MB-231 to the endothelium *via* forming stress fibers and focal adhesions with β1 integrin (Zhang and Lv, 2017). When suspended cells are injected to a mouse model, they showed significant increase in the metastasis to the lungs with more than double the mass in the lungs compared with cells that were grown in an adhesive environment (Figure 9) (Zhang et al., 2018). The survival of the suspended cancer cells was found to decrease with increasing shear level over time for MDA-MB-231, MDA-MB-468, and MCF-7 cells (Xin et al., 2019). Cells that survive these conditions were proposed to be responsible for generating metastatic tumors. These cells had unique morphology when replated, displayed lower stiffness, and were proposed to be a hybrid of epithelial/mesenchymal phenotype. Inhibiting actomyosin blocked flow-induced cell death, whereas activating actomyosin decreased cell survival. Moreover, cells with altered mechanical properties that survived shear in suspension were chemoresistant to the chemotherapy drug, fluorouracil, suggesting a connection between drug resistance and flow mechanotransduction. With more evidences that show flow shear may result in chemoresistance, e.g., to paclitaxel (Novak et al., 2019) and doxorubicin (Triantafillu et al., 2019), more holistic testing may be required to add mechanical signals such as flow to the current 2D monolayer high throughput screening test.

Flow mechanotransduction is altered in cancer cells and potentially also regulates cell metabolism. Fluid flow in 3D interstitial flow environment caused increased activity of AMP-activated protein kinase (AMPK), with MDA-MB-231 being more sensitive than MCF-10A (Steele et al., 2019). AMPK is recognized as a tumor suppressor and is activated when cell energy is low as based on ratios of ATP and other metabolic molecules. The AMPK activation data in cancer have led to the hypothesis that the AMPK effect can be location-dependent, e.g., flow caused AMPK activation localized to mitochondria and inhibiting FAK and Src removed this effect. Further providing evidence on the location dependent AMPK response in MDA-MB-231 to fluid shear was reported including plasma membrane, cytosol, nucleus, mitochondria, and Golgi apparatus (Guo et al., 2020). It was also observed that myosin II, a contractile protein, was required for mitochondrial AMPK mechanotransduction under flow and fluid shear-induced cell migration.

The research has largely concentrated on tumor cells and their response to mechanical signals. For a more holistic measure, the role of surrounding cells and other cell phenotype transitions may need to be considered. Cancer associated fibroblasts may be generated through the endothelial to mesenchymal transition (EndMT). Human umbilical vein endothelial cells (HUVEC) underwent EndMT dependent on the stiffness of 3D collagen matrix and glycosaminoglycan (GAG) concentration, which process was accelerated by low levels of shear flow (Mina et al., 2017). GAGs are important components of glycocalyx shear sensing which may be activated in both endothelial cells and cancer (Tarbell and Cancel, 2016). It was shown that shear stress activated GAG cell surface receptors and GAG synthesis while decreasing breast cancer spheroid size. As a result, shear stress significantly activated the interaction of endothelial and breast cancer cells, thus increasing cancer cell migration rate, traveled distance, and proliferation. In this model, the mechanically active tumor environment activated endothelial EndMT, which further promoted cancer cell growth and metastasis.

Endothelial cell surface receptors can also be activated by the shear flow environment. Such activation enables endothelial remodeling and also may be used by cancer cells for attachment and migration. Shear flow in the range of that experienced in a medium-sized vessel increased the attachment of MDA-MB-231 to HUVEC (Gomes et al., 2003). Simulating the inflammation in a tumor environment, TNF-α treatment of a microvessel network also caused HUVEC layer permeability resulting in a 2.3-fold increase of cancer transmigration rate (Chen et al., 2013). In addition to factors like TNF-α, migrating cancer cells in vasculature can interact with E-selectin on endothelial cell surface and soluble E-selectin shed in shear environments (Kang et al., 2016). Soluble E-selectin promoted the migration and adhesion under fluid shear for MDA-MB-231 and MDA-MB-468 cells both of which are CD44^+/high^. In contrast, CD44^−/*low*^ cells, MCF-7 and T-47D, had negligible response to shear related adhesion in the presence of soluble E-selectin. Enhancement of CD44^+/high^ adhesion activated by E-selectin was abolished with FAK inhibition. Furthermore, adhesion of MDA-MB-231 treated with E-selectin caused permeabilization of the endothelium which could aid migration out of the vasculature. These effects were demonstrated in a mouse model when tumor cell homing to lungs was increased 2.5-fold when treated with E-selectin. To prevent such a process from occurring, an E-selectin targeted aptamer (ESTA) may be developed. An ESTA was found to inhibit shear related adhesion of MDA-MB-231 and MDA-MB-468 to endothelial cells, and the results were confirmed with *in vivo* ESTA injection resulting in a 92% reduction in metastasis of 4T1 breast cancer cells (Kang et al., 2015). A continued study of endothelial activation, receptor profiles, and fluid shear response will be required to understand multiple aspects of the metastatic cascade. Table 2 shows some representative concepts/findings of the mechanical loading milieus (stretch, compression, fluid shear) control of breast cancer cells.

## Potential Mechanotransduction Mechanisms

Studies on environmental cues and mechanical loading signals have proposed mechanotransduction pathways involved in numerous aspects of the metastatic cascades of breast cancer cells. Identified pathways will be useful for suggesting treatment targets and also be used as diagnostic markers. Mechanotransduction elements and pathways have been described throughout this review. Some of the key pathways of breast cancer cell progression will be further highlighted in this section.

Starting at the cell periphery, plasma membrane proteins, glycocalyx or pericellular matrix, cell-ECM adhesion, and cell-cell junctions all have roles in cancer adaptations. ECM connections *via* transmembrane integrins are integrated into the cell at focal adhesions, which have complexes of physical anchoring proteins that can also be involved in mechanosignaling pathways. Elements associated with focal adhesion signaling and downstreams include FAK, vinculin, paxillin, talin, Src, PI3K, ERK, etc. Focal adhesion mechanotransduction is implicated in many processes involving strain sensing as was found in stretch or flow conditions. Each of these elements interacts with numerous pathways and connect with other mechanotransduction subsystems. For example, the focal adhesion protein talin connects integrins to F-actin, allowing force transmission and adaptation throughout the cell. Talin can be overexpressed in breast cancers and blocking talin could inhibit cancer invasion and migration (Wen et al., 2019). The talin-vinculin-actin filament linkage can transduce forces inside the cells and as a result regulate cancer metabolism. It was shown that through this focal adhesion linkage the mechanical signals activate the PI3K/Akt/mTor pathway causing oncogenic adaptation in cell metabolism and apoptosis resistance (Rubashkin et al., 2014). Further highlighting the interconnection of mechanotransduction pathways, PI3K also participates in the Rac1-JNK pathway which regulates cell motility and cytoskeletal adaptation (Carey et al., 2017). In addition to the results presented in previous sections including integrins, FAK, etc., clearly, focal adhesion-based mechanotransduction adapts to the mechanical environment, providing direct force transmission routes to other elements and further triggering cell processes involved in cytoskeletal adaptation, migration, and even metabolism and apoptosis.

Moving inwards, the cytoskeletons and associated regulators of cytoskeletal turnover and tension can mediate cell migration and adaptation processes. Of particular interests are Rac1 and RhoA which have opposing effects and locations in polarized migrating cells. Rac1 and RhoA are coordinated in breast cancer cell migration, so that Rac1 causes actin assembly at the leading edge of the cell migration and RhoA regulates cell contractility with more localized at the trailing edge (Byrne et al., 2016). In another view, migration driven by Rac1 has been reported to be mesenchymal in nature, whereas migration dependent on RhoA/ROCK is more amoeboid in nature and suited for migration through less dense matrices such as collagen. Targeting both Rac1 and RhoA/ROCK pathways may therefore be an effective strategy for preventing tumor cell invasion and metastasis (Jones et al., 2017).

Prevention of metastasis to bone targeting RhoA/ROCK pathway has been investigated considering Rho/ROCK has aberrant expression in breast tumors. Inhibiting ROCK decreased cell proliferation and metastasis to bone both *in vitro* and *in vivo* mouse model (Liu et al., 2009). Induced overexpression of ROCK in normally non-metastatic MCF-7 resulted in a metastatic phenotype, inducing metastasis to the hindlimbs and liver. The mass of the metastatic tumors was decreased by 77% with the application of ROCK inhibitor, Y27632. The context and local tissue architecture may need to be considered when interpreting such results, considering environment and dimensionality influence cell behavior and mechanotransduction pathways as described earlier. ROCK was reported to be elevated in T4-2 cancer cells but not in non-malignant S1 cells, but such a difference was only observed in 3D but not in 2D culture (Matsubara and Bissell, 2016). Since ROCK acts through cytoskeletal tension signaling, related cytoskeletal elements can also play a role. Increased myosin light chain (MLC) phosphorylation was found in malignant cells in accordance with unorganized F-actin; ROCK inhibition reorganized F-actin and repolarized the cells. As another element, it was shown that triple negative cells had low responsiveness to ROCK inhibition but this effect was reversed when cells were manipulated to overexpress E-cadherin, proposing a connection among cytoskeleton, ROCK activity, and E-cadherin cell-cell adhesion. Together, forces and mechanotransduction pathways are transmitted from anchorage points to the cytoskeleton, for both cell-matrix junction and cell-cell junction, and vice versa.

Mechanotransduction interfacing with the nucleus includes direct links which transmit forces to the nucleus and a host of mechanotransduction cascades that regulate genes. One route of mechanical information transfer is the translocation of proteins to the nucleus. As discussed above, YAP can translocate to the nucleus in response to the local strain environments, both static and dynamic. YAP and the transcriptional co-activator PDZ-binding motif (TAZ) are transcription factors typically associated with the Hippo pathway. These factors are particularly relevant for cancer research, since these have been implicated in the formation of cancer stem cells and EMT transition in response to mechanical forces (Jabbari et al., 2015). Some catenin subtypes are also able to translocate to the nucleus in response to mechanical signals, as is often coordinated with cadherin cell-cell junctions. In stem cells, the canonical Wnt/β-catenin pathway is involved in stem cell self-renewal. The canonical and non-canonical Wnt pathways also regulate cancer stem cell development and may provide a number of potential treatment targets for cancer stem cell elimination (Katoh, 2017). In breast cancer, β-catenin can be translocated to the nucleus in response to substrate stiffness which may drive tumor progression through altering microRNA expression (Mouw et al., 2014). Guerra et al. (2017) found that modification of cells with the ROCK inhibitor, fasudil, caused β-catenin translocation to the nucleus. ROCK inhibition decreased breast cancer migration and also activated the canonical Wnt/β-catenin pathway. Consequently, fasudil may be a candidate for preventing triple negative breast cancer metastasis and other treatments may be developed considering the role of translocation of proteins to the nucleus.

Finally, LINC complex-based nuclear mechanotransduction proteins, e.g., SUN1, SUN2, nesprin1/2, providing direct force links from the cytoskeleton to the nucleus may be important cancer markers, which are downregulated in breast cancer and may serve for diagnostic or prognostic purposes (Matsumoto et al., 2015). It was also recently reported that nesprin-2 and lamin A/C were significantly decreased in malignant MCF-7 and MDA-MB-231 cells when tested on patterned substrates while these proteins were increased in benign MCF-10A cells, suggesting that the downregulation of LINC complex and nuclear lamina may have direct influence on cancer state and invasiveness (Antmen et al., 2019).

## Perspective

Mechanobiology is now well-posed to solve some of the most pertinent issues in cancer detection, grading, and typing. In this review paper, we highlighted the effects of mechanical factors in oncogenesis, the premetastatic niche, the metastatic cascade, colonization of secondary metastatic sites, and dormancy and drug resistance. Mechanotransduction pathways have been identified in the regulation of these processes, potentially suggesting treatment targets. Also, by testing mechanical effect on drug resistance, therapies may also be improved to increase the efficiency of current treatments. Besides sensitizing cells to chemotherapy drugs, understanding how to modify aberrant microenvironments for drug penetration may also be attempted. These works will require more biomimetic tumor models to understand cancer cell sensing and response to environmental cues and mechanical loading as well as the interaction with cancer-associated fibroblasts, luminal cells, and vascular cells. This perspective takes the cancer as tissue or organoid approach. Extending the soil-and-seed paradigm in the cancer research to the current understanding of cancer complexity in multiple domains, cancer may no longer be thought of as just a seed that takes root in favorable soil. Cancer is more akin to a seed that acts as a terraforming agent, capable of modifying local and system soil ecology. This is particularly evident when considering the mechanical factors of tumor progression. Modified ECM stiffness, chemical composition and physical substrate pattern, and transformation of endothelial, MSCs, and immune cells to tumor-supportive roles *via* mechanical loadings and mechanotransduction pathways associated thus reinforce the importance of considering the mechanical domains in tumor progression. Since both malignant and cancer-associated fibroblasts have demonstrated mechanical memory effect, model systems that mimic multiple steps of the metastatic cascade will be particularly useful, for example, studying intravasation and adherent migration after cells are conditioned in compressive environments, or studying the colonization of secondary tumor sites after mechanical loading exposure that mimics the invasion process.

In addition to therapies involving mechanical signaling or mechanotransduction pathways, classification of cancer and more accurate prognostics would benefit from considering the mechanical domain. Large heterogeneities in mechanical response still exist within individual cell lines and among cells with the same immune-profile (HER, ER, PR, etc.) or classification (luminal A, claudin low, etc.). Mechanotyping using measures of cytoskeletal stiffness, nuclear deformability, traction force, and adhesion profile could supplement existing classification methods and may help resolve heterogeneities present in the current classification system. For example, topography-based assessments may be effective for identifying malignant cells and distinguishing these form benign cells. Assessment of morphology and migration on different topographies may be used to screen for multiple salient features in cell behavior in a relatively short time (Alvarez-Elizondo et al., 2020). Guck et al. (2005) were able to interrogate the deformability of human mammary epithelial cells rapidly using a microfluidic setup with optically-induced surface tension without direct cell contact. The utility of this strategy has been demonstrated in oral cancer diagnosis with a low optical stress applied in the range of 1–5 Pa (Remmerbach et al., 2009). Applying a similar concept, forcing cells from blood samples quickly through a microfluidic channel allows the identification of cell types and detection of pathological changes *via* rheological analysis (Toepfner et al., 2018). Taking this a step further, dynamic typing *via* the response to mechanical loading regimes could be attempted. The activation of key mechanotransduction regulators to mechanical loadings would supplement the mechanotyping and current surface marker methods. This may necessitate the development of high throughput mechanical loading systems for such testing. Expanding on this, a novel device for combining multiple loading regimes, compression, fluid shear, and stretch, to be even more physiologically relevant will be helpful for interrogating tumor organoids. Current systems are typically limited to simple loading regimens and may not capture the complex and dynamic mechanical loading that exists *in vivo*.

## Author Contributions

BR and JL: manuscript planning, writing, and revision. EK and TB: manuscript revision and writing. All authors contributed to the article and approved the submitted version.

## Conflict of Interest

The authors declare that the research was conducted in the absence of any commercial or financial relationships that could be construed as a potential conflict of interest.
